# Exploring Nearest Neighbor Interactions and Their Influence on the Gibbs Energy Landscape of Unfolded Proteins and Peptides

**DOI:** 10.3390/ijms23105643

**Published:** 2022-05-18

**Authors:** Reinhard Schweitzer-Stenner

**Affiliations:** Department of Chemistry, Drexel University, Philadelphia, PA 19104, USA; rs344@drexel.edu; Tel.: +1-215-895-2268

**Keywords:** unfolded proteins, isolated pair hypothesis, nearest neighbor interactions, chemical shifts and J-coupling, conformational entropy

## Abstract

The Flory isolated pair hypothesis (IPH) is one of the corner stones of the random coil model, which is generally invoked to describe the conformational dynamics of unfolded and intrinsically disordered proteins (IDPs). It stipulates, that individual residues sample the entire sterically allowed space of the Ramachandran plot without exhibiting any correlations with the conformational dynamics of its neighbors. However, multiple lines of computational, bioinformatic and experimental evidence suggest that nearest neighbors have a significant influence on the conformational sampling of amino acid residues. This implies that the conformational entropy of unfolded polypeptides and proteins is much less than one would expect based on the Ramachandran plots of individual residues. A further implication is that the Gibbs energies of residues in unfolded proteins or polypeptides are not additive. This review provides an overview of what is currently known and what has yet to be explored regarding nearest neighbor interactions in unfolded proteins.

## 1. Introduction

**Figure 1:**
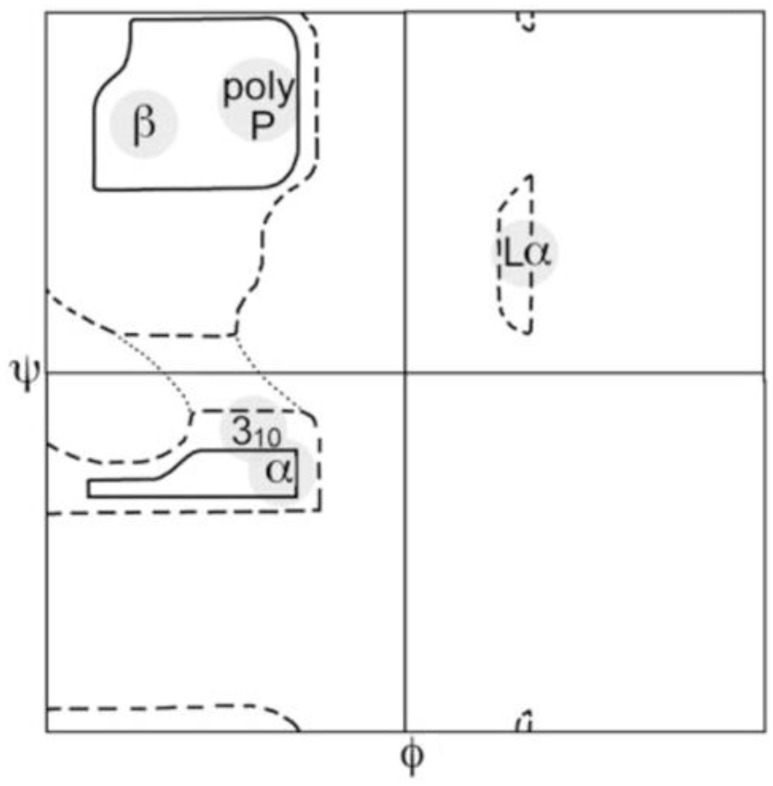
Sterically allowed *φ*, *ψ* space proposed by Ramachandran. Solid lines enclose the region allowed by hard-sphere bumps at standard radii; dashed lines show the region allowed with reduced radii; dotted lines add regions allowed if τ (N-C_α_-C′) is relaxed slightly. Ψ and *φ* values run from −180° to 180°. Taken from [29].

For a long period of time the unfolded states of proteins have not attracted considerable interest in the protein community because their basic properties seemed to be obvious and generic. Following early models of Flory, it was assumed that unfolded peptide chains are describable by the random coil model irrespective of its amino acid residues sequence [1,2,3]. Locally, it was assumed that the latter sample the entire sterically allowed region of the Ramachandran plot, which is rather similar for different amino acid residues, glycine and proline being the exceptions (cf. Figure 1). Hence, the unfolded state has been thought to be just a reservoir of conformational entropy that has to be overcome for a protein to fold.

This view has changed over the last 25 years for a variety of reasons. First, the discovery of intrinsically disordered proteins (IDPs) that perform biological functions revealed that the nature of their amino acid residues must play a role, since the results of bioinformatic analyses suggested that polar and charged amino acid residues occur more frequently in IDPs than they do in folded proteins [4,5,6,7]. IDPs are of particular relevance for cell signaling processes that frequently involve a transition between disordered and folded states. Intrinsically disordered segments of otherwise folded proteins are involved in pivotal protein–DNA and ligand–receptor interactions [5,8]. Some IDPs are of great biomedical relevance in that they are prone to self-assembly into amyloid fibrils [9,10,11]. Canonical examples are the amyloid β-peptides Aβ_1-40_ and Aβ_1-42_, α-synuclein and the tau protein [12,13,14].

The second reason for the growing interest in unfolded proteins and peptides is based on the discovery that, contrary to Flory’s assumption and a belief cultivated over decades, individual amino acid residues do sample a space less than the one dictated by steric constraints. Moreover, experimental data and coil library analyses revealed that the Ramachandran distribution depends very much on the nature of the amino acid side chains [15,16,17,18,19,20,21,22,23]. Multiple lines of evidence lead to the conclusion that the amino acid residue specificity of conformational distributions is due to specific backbone/side chain–water interactions [24,25,26,27,28]. All these results suggest the thermodynamics of unfolded proteins and IDPs are more complicated than originally anticipated.

The third reason for the increasing attractiveness of unfolded proteins is that they do not always behave like an ideal random coil. While the scaling laws obtained for the radii of gyration and end-to-end distances of a large number of foldable proteins subjected to denaturing conditions (i.e., high urea concentration) indicate a nearly perfect self-avoiding random walk (i.e., an exponents close to ν = 0.59) [30], IDPs or disordered protein segments can exhibit scaling laws with exponents spreading over a large range, depending on the apparent net charge of the investigated sequence [31,32,33,34,35]. Exponents below 0.5 indicate a compact structure, while values above 0.6 reflect extended coils preferably interacting with water [34]. It should be noted in this context that it is known for quite some time that proteins such as cytochrome c prefer some type of compact molten globule state under certain denaturing conditions (low or high pH, high temperature, on membrane surfaces, even in the presence of urea and guanidine hydrochloride at neutral pH) [36,37]. Interestingly, even scaling coefficients between 0.53 and 0.6, indicating ensembles somewhere between an ideal and self-avoiding random coil, do not exclude the possibility that the unfolded proteins exhibit local transient structures [38]. Calculations reported by Fitzkee and Rose revealed that an unfolded protein can behave like a self-avoiding random coil while still showing considerable local order [39].

Irrespective of the complexities indicated above one might arrive at the conclusion that if the intrinsic structural propensities of the amino acid residues are known, then the conformational sampling of an unfolded protein could be predicted, and its energetics and entropic content assessed. Different propensity scales are available in the literature, which could be used to this end [23,40,41,42,43,44,45]. However, such a modeling of unfolded states requires the absence of non-local contacts between the amino acid residues and the validity of Flory’s isolated pair hypothesis (IPH), which states that the conformational dynamics of residues do not correlate. If both requirements were met residue energetics and entropies would be additive. Unfortunately, none of the above conditions are generally met. While non-local contacts might be negligible in extended IDPs (solvation energy dominates over intrapeptide interactions), several lines of evidence suggest that the isolated pair hypothesis is not valid irrespective of the specific coil state of the protein.

While the influence of non-local contacts on the compactness and local order of IDPs and unfolded proteins has been explored in some detail [14,46,47,48,49], work on nearest neighbor interactions (NNIs) appears somewhat fragmentized. It is the goal of this review to provide an overview over the rather diverse work performed over the last 25 years that was aimed at exploring how NNIs affect conformational propensities of amino acid residues. We will compare the results of these efforts and shed some light on their relevance for a thorough understanding of unfolded and disordered proteins.

Even though the term random coil is clearly defined in the polymer literature (as nicely delineated in [50]), the term is often used as a label for unfolded and disordered proteins in an indiscriminate way. Strictly speaking, the term can only be used for sufficiently long polymers comprised of rigid monomers (peptide groups) and freely rotatable linkers, the length dependence of which can be described by a power law for its mean radius of hydration; i.e., $\langle R_{h}\rangle \sim N^{\nu }$, where *N* is the number of peptide units. As indicated above, the exponent is 0.59 in a good solvent. Locally, the random coil model is based on the assumption that amino acid residue conformations are restricted solely by steric hindrances between the side chains as well as between side chains and backbone groups and by electrostatic interactions [51]. However, as indicated above, peptide/protein-solvent interactions reduce the available conformational space further and increase the anisotropy of the residue orientations [24,25,27,28,52,53,54,55]. Moreover, unfolded and disordered proteins frequently contain segments that can transiently adopt regular secondary structures [14,38,49,56]. Hence, real unfolded polypeptides and proteins do not meet the requirements for a random coil on a local level while they still might do so on a more global level [30,39,57]. Therefore, we follow the late Harold Scheraga in that we use the term statistical coil instead, which correctly reflects the fact that different chain conformations differ in terms of their Gibbs free energies [56,57].

This review is organized as follows. In the first step some basic thermodynamic aspects of NNIs are delineated. The main part of the review is divided into three sections. Part 1 provides some general description of the statistical thermodynamics of NNIs. This is followed by Part 2, in which we review the coil library analyses that extracted the conformational propensities and NNI effects from the structure of the denatured proteins. In Part 3, we provide an overview of the computational studies that explored the underlying physical mechanism of NNIs. Part 4 describes the experimental studies, mostly on short peptides, that were specifically aimed at quantifying the NNIs between different types of residues. A summary and outlook finish the review.

## 2. Thermodynamic Aspects of Nearest Neighbor Interactions

It is now well established that the Ramachandran plot of amino acid residues in unfolded peptides and proteins contains several basins associated with different secondary structures. The most prominent ones are shown in Figure 1. Generally, based on experimental studies with blocked dipeptides and unblocked GxG guest–host tripeptides (x: host amino acid residue), all residues predominantly populate two basins in the upper left part of the Ramachandran plot, which are assignable to polyproline II (pPII) and β-strand (β) (between 70 and 100%) [23,40,42,43]. The basin of the former can be found in a region between *φ*-values of −90° and −60° while the *ψ*-values might vary between 130° and 180°. The center of β-strand basins can vary over a range between *φ*-values of −100° and −140°. Corresponding *ψ*-values vary again between 130° and 180°. This area encompasses backbone structures associated with different types of β-sheet structures. The sampling of other basins by residues in the above peptides is somewhat more limited. The most prominent one is assignable to right-handed helical structures (−70° < *φ* < −30°, −60° < *ψ* < −20°), which encompasses canonical α-helical and 3_10_-like conformations. Quite a few residues sample the region of γ (30° < *φ* < 70°, −80° < *ψ* < −50°) and inverse γ-turns (*φ*, *ψ* with opposite sign). In specific cases residues were found to sample left-handed helical structures (30° < *φ* < 70°, 20° < *ψ* < 60°), and conformations in the bridge region around *φ* = −60° and *ψ* = 0, which are generally found at the i + 2 position of type I and II’ β-turns [43,45,58,59]. In addition, aspartic acid and asparagine residues can form asx-turns, supporting the conformations (50° < *φ* < 80°, 120° < *ψ* < 180°). Asx-turns consist of a sequence of three residues, the first of which is either aspartic acid or asparagine [60].

The population $\chi_{i,k}$ of these conformations can be calculated as

(1) $$\chi_{i,k}=\frac{e^{G_{i,k}/RT}}{\sum_{k}e^{G_{i,k}/RT}}$$

where *G_i,k_* is the Gibbs free energy if the *k*-th conformation of the *i*-th residue. *R* is gas constant and *T* the temperature in Kelvin. It can be decomposed as follows:

(2) $$G_{i,k}=G_{i,k}^{0}+\delta G_{ij,k}^{0}+\delta G_{ij,kl}$$

where $G_{i,k}^{0}$ is the Gibbs energy of the *k*th conformation of the *i*th residue in the absence of nearest neighbor interactions, $\delta G_{ij,k}^{0}$ is the Gibbs energy contribution by the *j*th neighbor that reflect the steric and physico-chemical properties of the neighbor irrespective of its adopted conformation and $\delta G_{ij,kl}$ is the conformation-dependent contribution of the *j*th neighbor in its *l*th conformation.

Distinguishing between these two contributions to the NNI–Gibbs energy is technically difficult (*vide infra*) but nevertheless of conceptual significance. If the NNI was comprised only of $\delta G_{ij,kl}^{0}$ contributions, there would be no cooperativity between the interacting residues. In such a case, which is comparable to the first-order contribution to the eigenenergies of quantum mechanical systems, the internal energies, entropies and thus also the Gibbs energies of residues in an unfolded polypeptide would still be additive and thus the isolated pair hypothesis would still be valid. Therefore, only conformation dependent NNIs are a real game changer in that the population of a certain conformation of a considered residue becomes dependent on the conformation adopted by its neighbors. To illuminate the significance of conformation dependent NNIs, let us consider an oligopeptide with only two interacting residues. In the absence of any cooperativity, the mole fraction of a conformation pair *k*, *l* is calculated as

(3) $$\chi_{kl}=\frac{e^{\left({G^{\prime }}_{1,k}+{G^{\prime }}_{2,l}\right)/RT}}{\sum_{k}e^{{G^{\prime }}_{1,k}/RT}\sum_{l}e^{{G^{\prime }}_{2,l}/RT}}$$

where the Gibbs energy terms *G*′_1,*k*_ and *G*′_2,*l*_ contain the first two terms of Equation (2). In the presence of cooperative NNIs, the corresponding equation for *χ_kl_* looks rather different:

(4) $$\chi_{kl}=\frac{e^{\left({G^{\prime }}_{1,k}+{G^{\prime }}_{2,l}+\delta G_{12,kl}+\delta G_{21,kl}\right)/RT}}{\sum_{k}\sum_{l}e^{\left({G^{\prime }}_{1,k}+{G^{\prime }}_{2,l}+\delta G_{12,kl}+\delta G_{21,kl}\right)/RT}}$$

where we consider the mutual influence of residue 2 on 1 and residue 1 on 2 in the numerator. The partition sum in the denominator runs over all peptide conformations, which is different from the product of partition sums of individual residues in Equation (3). Obviously, the corresponding conformational Gibbs entropies of the two systems are also different, namely,

(5) $$S=-R\left[\underset{k}{\sum }\left(\chi_{1,k}ln\chi_{1,k}\right)+\underset{l}{\sum }\left(\chi_{1,l}ln\chi_{1,l}\right)\right]$$

in the absence and

(6) $$S=-R\underset{k}{\sum }\underset{l}{\sum }\left(\chi_{kl}ln\chi_{kl}\right)$$

in the presence of cooperativity.

The theory introduced thus far solely considers the possibility that NNIs affect the Gibbs energies of residue conformations. However, it is equally likely that NNIs change the equilibrium position of a basin. This can be seen from a simple example. Assume that the potential function associated with a basin can be approximated by a harmonic potential of the type $V\left(q\right)=1/2\cdot k{\left(q-q_{0}\right)}^{2}$ where *q* represents one of the two dihedral coordinates. If the corresponding residue is subjected to a perturbing potential V(q)=aq+b, the equilibrium position shifts from *q* = *q*_0_ to $q_{0}^{\prime }=a/k+q_{0}$. If the *q*-dependence of the perturbing potential is more complicated, that expression for *q*_0_′ becomes more complicated as well.

In what follows, we will have to keep the theoretical thoughts of this section in mind when we go over the NNI literature.

## 3. Coil Library Studies

**Figure 2:**
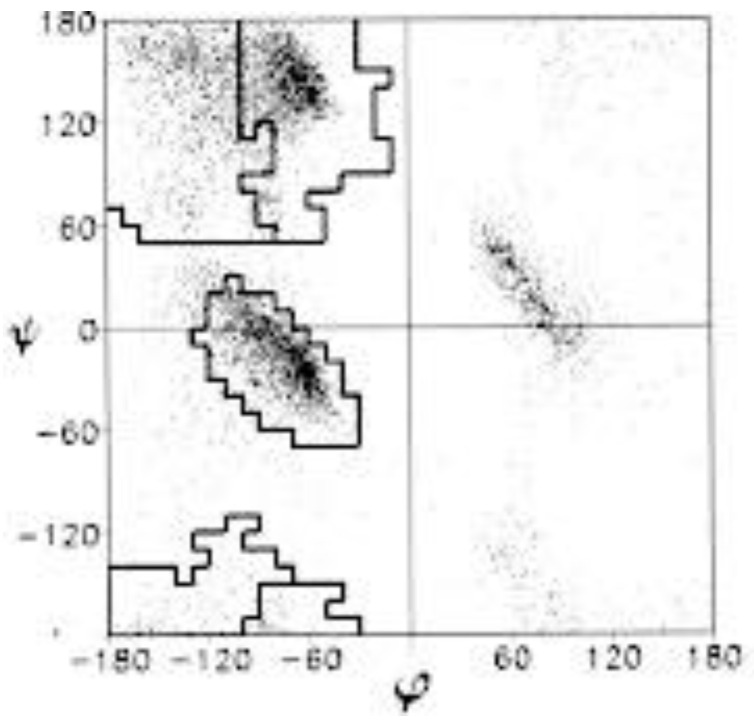
Ramachandran distribution of the sterically allowed region of amino acid residues in coil libraries. The two regions in the upper left and lower left quadrant are associated with β-strand and polyproline II (p), respectively. The region crossing the *ψ* = 0 line in the left part of the plot contains right-handed helical and turn-supporting conformations. Taken from [15] and modified.

In 1995, two important articles reported what the authors called conformational propensities of amino acid residues in coil regions of proteins. Swindells et al. determined the Ramachandran distributions of amino acid residues in coil regions of 85 structures obtained from the protein data bank [15]. They excluded any residues incorporated in right-handed helices and β-sheets to eliminate local and non-local interactions associated with the stabilization of these secondary structures. Moreover, they assumed that the thus obtained Ramachandran distributions could reflect intrinsic structural propensities and thus be representative of the respective conformational distributions in unfolded/denatured proteins. This conceptual model is based on the assumption that non-local interactions in these coils are random in nature and averaged out by considering a sufficiently large data set. Contrary to one of the basic assumptions of Flory’s random coil model, they found significant differences between the population distributions of different amino acid residues. They reported integrated propensities for four regions of the Ramachandran plot shown in Figure 2. If one converts their propensity values into fractions by dividing the reported propensity values by their sum, one obtains 0.35, 0.21 and 0.33 for the pPII, β-strand and right-handed helical region, respectively, of alanine. For valine, however, the corresponding fractions are 0.24, 0.45 and 0.22. Swindells et al. found that the obtained propensity values correlated with secondary structure propensities for the β-strand while the correlation with right-handed helical propensities was found to be weak [15].

**Figure 3:**
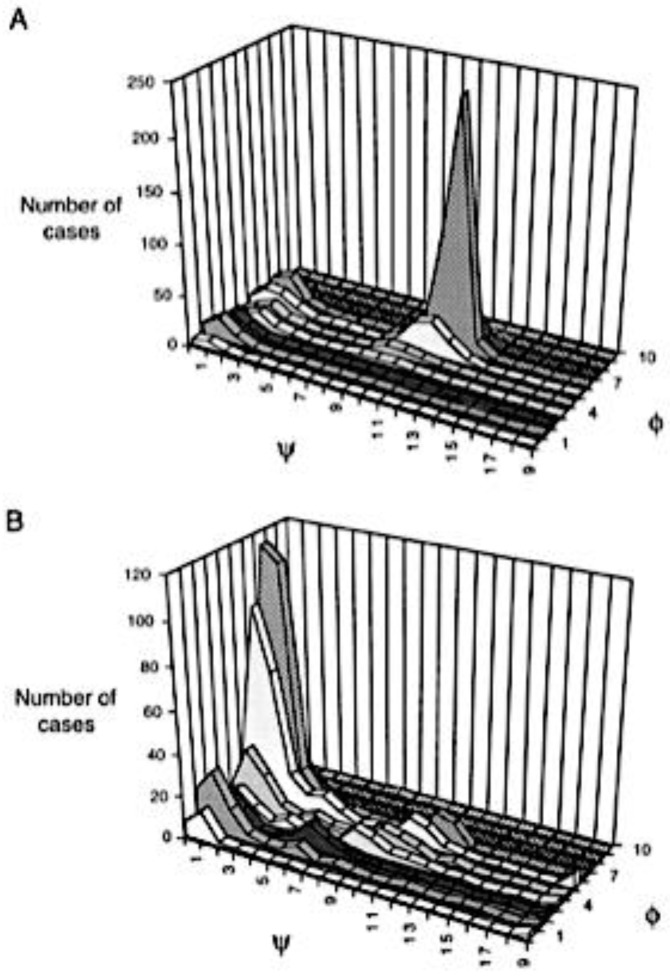
Conformational distribution of alanine residues inferred from the protein data sets. (**A**) The complete data set including residues in well-defined secondary structures. (**B**) Only alanine residues in coils outside of right-handed helical and β-sheet structures were considered. Each number on the abscissae represents an 18° interval starting at −180°. Taken from [16] with permission, 1995, Academic Press.

In earlier days, Ramachandran plots were indiscriminately derived from protein data sets because of the belief that all non-intrinsic influences could be averaged out by a sufficiently large data set, irrespective of its content of regular secondary structures. That this is not the case was demonstrated by Serrano [16]. Figure 3 shows the distribution of alanine obtained from an unrestricted data set (Figure 3A) and from a restricted coil set (helices and where sheets were omitted (Figure 3B). The difference is striking. While the former is dominated by an intense peak in the right-handed helical region the latter becomes maximal in the pPII region. Hence, Serrano’s data clearly reveal the propensity of alanine for polyproline II six years before experimental results obtained with an hepta-alanine peptide suggested the same and triggered a highly controversial debate. Details of this debate are summarized in an earlier review of Toal and Schweitzer-Stenner [22]. Serrano’s analysis, which was augmented by a comparison of the chemical shifts and ^3^J(H^N^H^Cα^), observed for short model peptides and calculated for coil library distributions, clearly corroborated the notion that different amino acid residues have different intrinsic propensities for specific conformations.

Nearest neighbor effects were first considered in a third paper by Penkett et al. that appeared in 1997 [61]. Keeping in mind the well-stablished fact that the helical and β-sheet propensities of amino acid residues are context dependent they were wondering whether such context dependencies would also be observable in the unfolded proteins for which they assumed minimal non-local interactions. To this end, they used ^1^H NMR to determine the ^3^J(H^N^H^Cα^) coupling constants for a 130-residue fragment of the fribronectin-binding proteins from *Staphyloccus aureus*. They observed that 9 of 16 glutamic acid residues preceded by residues with either branched or aromatic side chains exhibited coupling constants between 6.2 and 7.0 Hz, while the other 7 with asparagine or glutamate as upstream neighbors lie between 5.7 and 6.3 Hz.

Further insight into the underlying NNIs came from the average ^3^J(H^N^H^Cα^) values calculated for the coil library distributions of Swindells et al. [15]. These calculations were performed with a Karplus equation that relates the coupling constant to the dihedral backbone angle *φ* as follows [58]:

(7) $$J\left(x,y,\eta \right)=Acos^{2}\left(\eta +\theta_{1}\right)+Bcos\left(\eta +\theta_{2}\right)+C$$

where *x* and *y* denote the interacting nuclei *x* and *y*, η = *φ*, *ψ*, and θ*_i_* (*I* = 1, 2) are phase angles. *A*, *B* and *C* are empirical Karplus parameters obtained by fitting Equation (9) to the J-coupling data sets obtained for proteins with a known crystal structure [59,62,63,64,65]. Alternatively, these parameters could be obtained with density functional theory calculations but this has been accomplished thus far only for alanine [66]. It is likely that the exact parameters are side-chain dependent. Empirical values should therefore be considered as an average. In the case of unfolded and disordered proteins and peptides, the measured Karplus parameters represent a conformational average:

(8) $$\langle J\left(x,y\right)\rangle =\frac{\sum_{i=1}^{N}J\left(x,y,\eta_{i}\right)P\left(\eta_{i}\right)}{\sum_{i+1}^{N}P\left(\eta_{i}\right)}$$

where *P*(η*_i_*) is the probability for the residue to adopt a dihedral angle *φ_i_* or *ψ_i_*.

**Figure 4:**
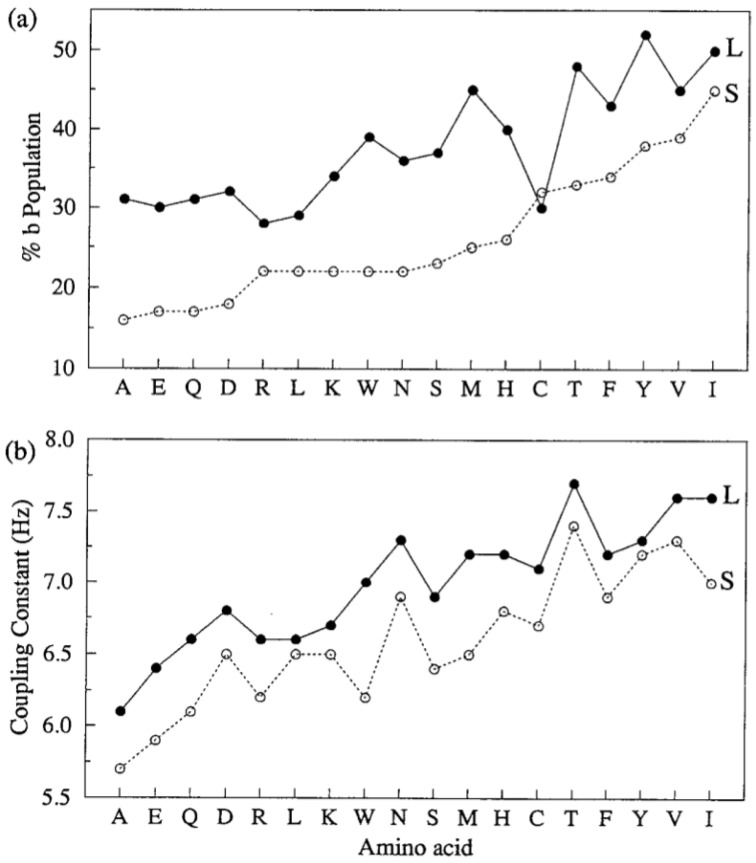
(**a**) Population of the extended β-region (b-region in Figure 2) by the indicated amino acid residue in the presence of an L-type (F, H, I, TV, W, Y) or S-type (remaining amino acid set, G excluded) upstream neighbor. (**b**) Corresponding average ^3^J(H^N^H^Cα^) values (Equations (6) and (7)). Taken from [61] with permission, 1997, Academic Press.

The results of the J-coupling analysis of Penkett et al. are shown in Figure 4 [61]. The average ^3^J(H^N^H^Cα^) values of 18 amino acid residues can apparently be subdivided into two groups. Residues preceded by F, H, I, T, V, W, and Y (L-group) exhibit systematically higher ^3^J(H^N^H^Cα^) values than corresponding residues preceded by representatives of the complementary group (G excluded). These results seem to indicate that sterically demanding aliphatic and aromatic residues move the overall distribution to lower (more negative) *φ* values. While important as a first data-based insight regarding the occurrence of NNIs, the results can be interpreted either as reflecting shifts of basins associated with different secondary structures and as redistribution between different basins. Experimental results to be discussed below reveal that NNIs can indeed cause both.

In what follows, we will focus on two different coil library analyses reported by the Sosnick and Dunbrack group, which both deal with NNIs in explicit terms [19,67]. Other reported libraries put a focus on individual propensities and conformational sampling, which is of lesser interest in this context [15,16,68,69]. Jha et al. conducted a very comprehensive coil library analysis of 2020 chains with more than twenty residues [18,67]. The authors produce Ramachandran plots for four different data sets: one set with no restrictions (all secondary structure sequences included), a second one from which helices and sheets were taken out, a third one for which turns were taken out as well and a fourth one from which flanking residues were also eliminated. The authors’ analysis clearly showed that taking helices and sheets out of the data set produce rather different Ramachandran distributions. Moreover, their analysis yielded rather different Ramachandran distributions for individual residues and revealed significant nearest neighbor influences.

**Figure 5:**
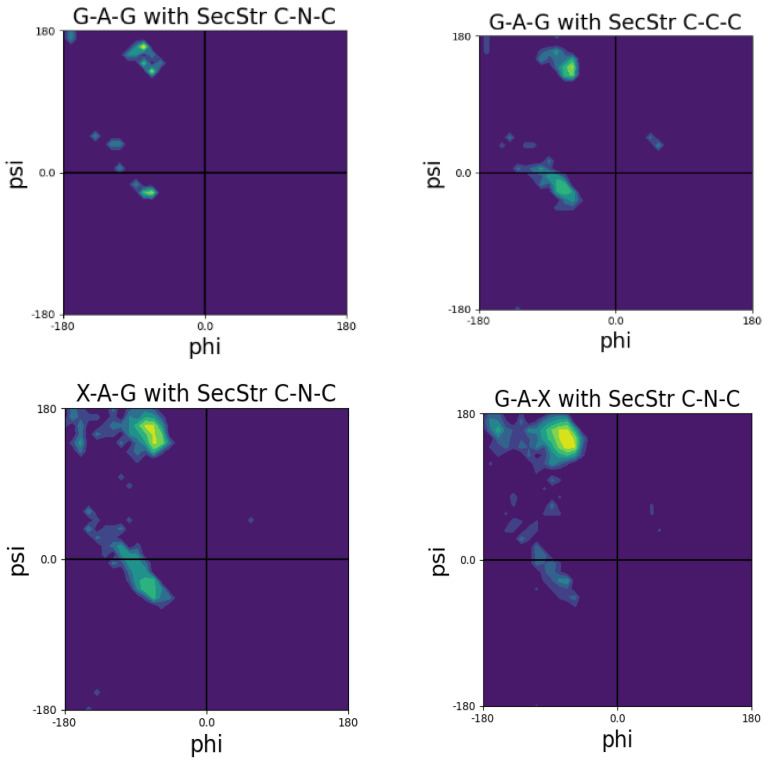
Ramachandran plots of the central residue in the indicated tripeptide sequences. Upper panel: (**Left**) only alanine residues in coils were considered; (**Right**) only alanine residues outside of helices and β-sheets were considered. Lower panel: X indicates the summation over all nearest neighbors outside of helices and β-sheets. The plots were directly taken from the website of the Sosnick group [70].

The number of residue data in the coil library of Jha et al. do not allow for Ramachandran plots of different tripeptide sequences to depict enough data points for most of the twenty amino acid residues. This is not surprising because a coil analysis excludes at least all secondary structures. Figure 5 shows Ramachandran plots for the central alanine residues for the following tripeptide sequences: GAG, with A taken from a disordered-like subset (labeled as ‘unfolded’ on the Sosnick group web site) and the two glycines from segments outside of helical and sheet structures; GAG with G and A data taken from segments outside of helical and sheet segments; and GAX and XAG, where X indicates an integration over all neighbors.

A comparison of these plots is quite revealing. The distribution of glycine-flanked alanine residues located outside the regular secondary structures seems to have comparable propensities for pPII and right-handed helical structures. The population of β-sheets is negligible. If one restricts the selection of alanine to coil-like segments, then pPII seems to be preferred over helical structures (Figure 5, upper panel, right). However, the number of counts might be too low to tell. If one integrates either over all upstream or all downstream neighbors, then pPII becomes clearly dominant (Figure 5, lower panel). Regarding the latter, the β-strand slightly gains at the expense of right-handed conformations. These data show that in this coil library nearest neighbors predominantly increase the pPII propensity of alanine.

The Sosnick group utilized their coil library distributions to explore the chemically denatured state of apomyoglobin, ubiquitin, the SNase fragment Δ131Δ and eglin C, for which they tried to reproduce experimentally determined NMR-based residual dipolar constants [18]. Jha et al. did so with coil library-based Ramachandran distributions with and without specific nearest neighbor interactions. Residues were taken from regions that contain neither helices nor sheets nor turn conformations. NNIs were considered solely for residue dimers for which the total internal energy was written as

(9) $$U\left(a_{i},b_{i},a_{i+1},b_{i+1}\right)=U\left(a_{i},b_{i}\right)+U\left(a_{i+1},b_{i+1}\right)+\delta U\left(a_{i},b_{i},a_{i+1},b_{i+1}\right)$$

where *a_i_* labels the identity of the *i*th-residue that samples the basin *b_i_*. The interaction energy *δU* accounts for the cooperativity or anti-cooperativity between conformations *b_i_* and *b_i_*_+1_ of the two residues. It is related to the conditional probability *P*(*a_i_*,*b_i_*,*a_i_*_+1_,*b*_*i*+1_) by

(10) $$\delta U\left(a_{j},b_{j},a_{j+1},b_{j+1}\right)=-RT\left[\frac{P\left(a_{j},b_{j},a_{j+1},b_{j+1}\right)}{P\left(a_{j},b_{j}\right)P\left(a_{j+1},b_{j+1}\right)}\right]$$

**Figure 6:**
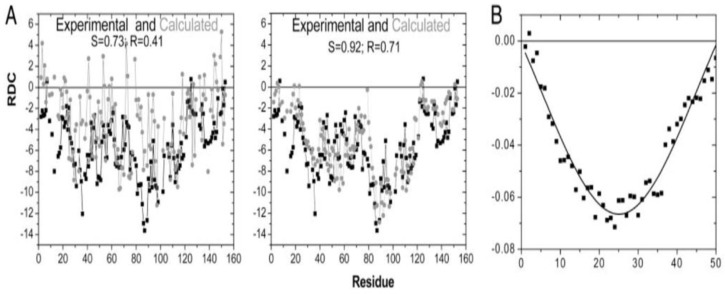
(**A**) Experimental (black) and calculated (grey) residual dipole coupling values for apo-myoglobin in 10% acrylamide. The employed model combined coil library data with MD simulations. **Left**: Calculations performed without considering NNIs. **Right**: Calculations performed with NNIs obtained from a comparison of Ramachandran plots. (**B**) Residual dipole coupling values of an idealized random coil ensemble of an A_50_ polypeptide generated without nearest neighbor coupling. Equal population (1/3) was assumed for pPII, β-strand and right-handed helical basins. Taken from [18] (free-access article).

The coil data basis of the authors was not large enough to allow for an empirical determination of NNIs. To gain information about the latter they performed Monte Carlo simulations with an energy functional for each basin with and without NNIs. Energy minimization was constrained by the utilized coil library distributions for individual residues. The energy functional did not contain any protein–solvent interaction; basically, the authors employed excluded volume effects. This procedure was carried out with and without nearest neighbor interactions. As one can infer from the results obtained for apomyoglobin, shown in Figure 6, calculations with NNIs achieved a much better reproduction of the experimental residue coupling constants. Equally interesting is the fact that the experimental values do not at all follow predictions based on an idealized random coil model (Figure 6B). The latter used three major isoenergetic basins for each residue of an A_50_ polypeptide (pPII, β-strand, right-handed helical) with a population of 0.33. The obtained V-shape reflect the decreased correlation with the molecular axis for residues closer to the termini. An analysis of the conformational ensemble of the investigated unfolded/denatured proteins reveals a dominance of what Jha et al. called stretched conformations in which individual residues sample predominantly pPII and β-basins. For the set of coil library residues used for the residual dipolar coupling analysis they obtained a mole fraction ratio of <pPII>:<β>:<right-handed helical> = 0.33:0.36:0.27. While the numbers seem to be reminiscent of a random coil supporting distribution (i.e., sampling of all sterically allowed regions), the very existence of distinguishable pPII and β-basins is not. Despite the deviation from an ideal random coil behavior on a local level, the radii of gyration calculated for the above and six additional unfolded/denatured proteins indicate self-avoiding random coils. This result is in line with computational results of Fitzkee et al., who showed that an ensemble of rods connected by flexible linkers can still reproduce the scaling law for self-avoiding random coils [39]. All these results show that it is necessary to distinguish between local and global aspects of random coils, as very early on emphasized by de Gennes [71] for polymers and by Toal and Schweitzer-Stenner for peptides and proteins [22].

While of great insight for an understanding of the relevance of NNIs, the above studies do not provide much specific information about how NNIs depend on the physico-chemical properties of the involved residues. The coil library analysis of Jha et al. suggests a strong anti-cooperativity between pPII and right-handed helical conformations of alanine and alanine-like as well as β-branched upstream neighbors, respectively. Aromatic residues positioned downstream seem to cause anti-cooperative interactions between the pPII states of the interacting residues [67].

An even more thorough and residue-specific analysis of coil libraries have been undertaken by Ting et al. [19]. Their data set contained 3038 proteins from the Uppsala Electron Density server. In line with the protocol of the Sosnick group they obtained different coil libraries by employing different restrictions regarding the selection of residues. The largest set contained loop residues (no regular secondary structure elements) for which all backbone atoms appear in the data set. Each residue is at least three residues away from the regular structures. This set was termed TCBIG, which contain the single letter designation of turn, coil, β-bridge, π-helix and 3_10_-helix. The second, reduced data set did not contain π- and 3_10_ helices (TCB). The authors classified their Ramachandran distributions in terms of the following five conformations (Table 1): α-helical, β-strand, polyproline II, left-handed helical and extended. Since considering all combinations of a given residue with its neighbors is an impossible task (20^3^ combinations) the authors confined themselves to selected ‘dimers’ where they changed the neighbor either upstream or downstream from the considered residue and averaged over all neighbors for the other side. If the influence of the two neighbors is not additive—a notion for which experimental evidence exist in the literature (*vide infra*)—the information obtained from Ramachandran plots of different pairs might not necessarily represent the NNIs between the pairs. In order to permit a thorough mathematical analysis of the obtained Ramachandran plots, the authors represented the latter by a continuous functional that can be ascribed to a combination of a two-dimensional Gaussian distribution located in close proximity to basins associated with secondary structures. While this modeling bears some similarity with the Gaussian model of Schweitzer-Stenner [72], differences should be emphasized. While the latter works with 1:1 assignments of Gaussians to the assumed basins of the Ramachandran plot (which would be five for the above set of conformations assumed by Ting et al.), the former functional is entirely based on the distributions of data points inferred from the coil library sets. In both cases, the functionals facilitate the mathematic analyses of distributions.

**Figure 7:**
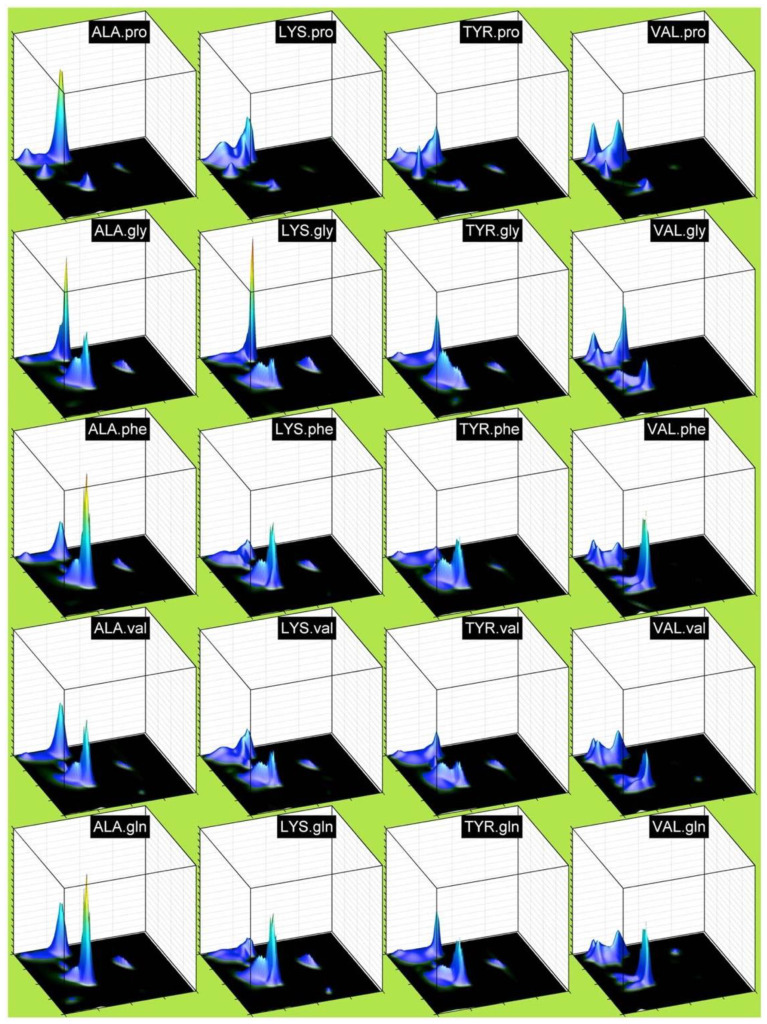
Ramachandran plots of alanine, lysine, tyrosine and valine in the presence of the indicated downstream neighbors. Distributions with different upstream neighbors were integrated. Taken from [19] (open access).

A closer look at the residue dimer distributions of Ting et al. reveal that some neighbors have a significant influence on specific residues. Let us again focus on alanine. Compared with X-AG, F, V and Q as downstream neighbors substantially increase the right-handed helical population of alanine at the expense of pPII (Figure 7). On the contrary, proline as a downstream neighbor stabilizes pPII. The nearest downstream neighbors of valine (including valine itself) increase the right-handed helical populations as well. While the latter is also significant in the coil library of the Sosnick group, the neighbor-induced helical population seems to be more pronounced in the Ting et al. library. This is a very surprising result. We consider the implications of these results below in the context of our discussion of NNIs in model peptides.

Ting et al. used Hellinger distances as a measure of dissimilarity between the Ramachandran plots. The latter can be calculated as

(11) $$H\left(P_{R}\left(\phi_{i},\psi_{i}\right),{P^{\prime }}_{R}\left(\phi_{i},\psi_{i}\right)\right)=||\frac{1}{2}\overset{\pi }{\underset{-\pi }{\int }}\overset{\pi }{\underset{-\pi }{\int }}\left(\sqrt{P_{R}\left(\phi_{i},\psi_{i}\right)}-\sqrt{P_{R^{\prime }}\left(\phi_{i},\psi_{i}\right)}\right)d\phi d\psi ||$$

where *P_R_* and *P*′_*R*_ are the two Ramachandran distributions to be compared with each other. An H value of zero means that the two distributions are identical, whereas a value of 1 indicate they are orthogonal. However, even very dissimilar Ramachandran distributions will not be able to produce values close to 1, because they cover only a limited fraction of the Ramachandran space. Schweitzer-Stenner and Toal employed the following criteria: H values between 0 and 0.1 indicate that two distributions are similar [73]. Values between 0.1 and 0.25 as well as between 0.25 and 0.4 indicate that they are modestly similar and dissimilar, respectively. Values above 0.4 reflect very dissimilar distributions (note, that Ting et al. multiplied their H-values with 100). Since the Hellinger distance is practically a measure of orthogonality, it is more sensitive to changes of basin position than it is to the redistribution of populations [73].

Table 1 lists the Hellinger distances for pairs of eight amino acid residues. The left values represent the H-distances for pairs irrespective of their neighbors (Ramachandran plots for different neighbors were added up) whereas the right values represent H-distances if Q is present as an upstream neighbor. Only a few residue pairs fall in the category ‘modestly dissimilar’. They mostly contain valine and asparagine. If only Q is considered as upstream neighbors, then all H-values are in the similar or modestly similar range. The significance of these values is not entirely clear. The integration over all neighbors might hide a strong influence of a few residue types. Valine and asparagine seem to be good candidates, as is, most likely, proline (values for P are reported by Ting et al.). We will return to the use of Hellinger distances below when we discuss investigations of short peptides in water.

## 4. Simulations

The first thorough computational investigation of NNIs was carried out by Pappu et al. [68]. The authors confined themselves on exploring the interactions between alanine residues in a blocked oligo-alanine peptide. The authors sub-divided the Ramachandran plot into 6 × 6 equally sized mesostates (60° × 60°). Their Monte-Carlo calculations were performed with a rather simple hard sphere model by means of which they just explored the sterically available space, very much in line with the classical Ramachandran approach [69]. They identified clashes between nearest neighbors sampling mesostates in the right helical region, while nearest neighbors sampling mesostates in the pPII and β-strand regions do not interfere with each other. Their results led the authors to conclude that an increasing chain length (of their oligo-alanine peptide) leads to a reduction in conformational space in the unfolded state, which reduces the conformational entropy and thus facilitates the folding into an overall right-handed α-helical conformation.

**Figure 8:**
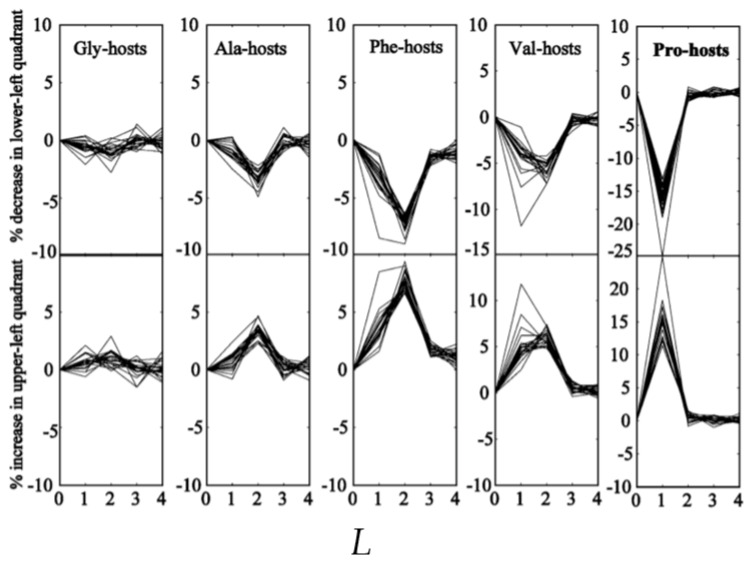
Changes in the propensity of 22 different guest residues (20 natural amino acid residues, norvaline and norleucine, each of which represented by a curve) for conformations in the lower and upper left quadrant of the Ramachandran plot as a function of the increasing length L of the oligopeptide. The host guest system is defined in the text. Taken from [74] with permission, 2005, American Chemical Society.

In a subsequent paper, Tran et al. investigated how steric-based NNIs depend on different type of neighbors [74]. They explored the conformational propensities of 22 amino acid residues (norvaline and norleucine in addition to the natural ones) in N-acetyl-(host)_L_-x-(host)_L_-N-methylacetamide (L: number of host residues) host–guest blocked tetrapeptides. Glycine, alanine, valine, phenylalanine and proline were selected as hosts. The result of their analysis is shown in Figure 8. While the influence of glycine on the guest residue is negligible (as one would expect), all other hosts (including proline) shift conformational sampling from the lower (all types of right-handed helical conformations) to the upper left quadrant (pPII and all types of β-strand). Interestingly, the underlying NNIs seem to be more pronounced for L = 2 (influence of nearest and second nearest neighbor) for A, F and, in part, V hosts. Altogether, the NNIs identified by Tran et al. produce more stretched peptide and protein conformations in the unfolded state than expected based on Ramachandran-type distributions. In that regard, their results are at variance with the nearest neighbor effects inferred from the coil library of Ting et al. [19]. However, for an increasing chain length, conformational entropy causes the chain to depart from a rod-like structure. Consequently, longer polypeptides and denatured proteins still obey the scaling law for a self-avoiding random coil.

NNIs also played a role in the MD simulations of Gnanakaran and Garcia on oligo-alanine peptides of a different length [75]. These authors used a modified Amber force field (A94 mod) for which they eliminated the force constants for the two dihedral backbone angles. They found that the pPII conformation of the residues is stabilized by NNIs that involves the optimal packing of water molecules in a groove formed by the peptide backbone of at least four residues [27]. However, if the number of alanine residues exceeds ten [27], helical conformations stabilized by intrapeptide hydrogen bonding become more likely, in agreement with experimental results. While the results of this work are important due to its emphasis on the role of the solvent, the elimination of the intrinsic force constants for backbone dihedral angles seems to be somewhat heuristic.

**Figure 9:**
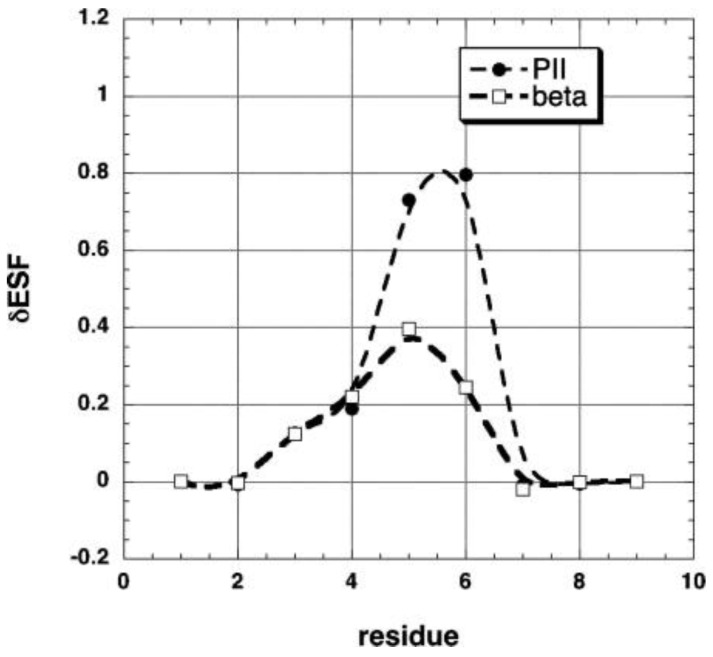
Changes in the electrostatic solvation free energy as a result of substituting alanine at position 5 of an A_9_ peptide by valine. The free energy is expressed in units of kcal/mol. Taken from [76] (open access).

Solvent effects also play a prominent role in the work of Avbelj and Baldwin [76]. These authors explored the electrostatic interactions between atoms within a residue and found them to stabilize the extended β-strand structures. pPII is stabilized by water in that it substantially shields this interaction. Such shielding effects get involved in NNIs, in that the solvation of residues depend on the conformation of neighbors. This is illustrated in Figure 9, which displays the change in the electrostatic solvation free energy caused by replacing an alanine at position 5 of a nine-residue oligoalanine peptide by a valine. The change is more pronounced if the valine residue adopts pPII than the one in the residue’s β-strand conformation. Moreover, the graphs in Figure 9 reveal the concomitant reduction in the electrostatic solvation energy of the neighbors, which particularly affects the downstream neighbor. Moreover, it is stronger for the pPII than it is for the β-strand conformation of valine. This important result suggests a cooperative interaction between the pPII state of valine and the β-strand conformation of the neighbor. Besides valine, Avbelj and Baldwin investigated the influence of the remaining 18 amino acid residues. They found the decrease in the electrostatic solvation free energy of the guest residue (compared with alanine) is particularly pronounced (>6.2 kJ/mol for pPII) for aromatic and aliphatic/β-branched residues (V, I, W, Y, F, H and T) and always larger if the guest residue adopts pPII. This work reveals that NNIs between residues adopting conformations in the upper left quadrant of the Ramachandran space are mostly solvent mediated.

The group of Sosnick has substantially contributed to our current understanding of NNIs. Besides their work on coil libraries [18,77], they conducted a thorough MD study on xAA and AAx tripeptides in water where x denotes the guest residue. To this end they employed three force fields in implicit water: Amber 94, the modified Amber force field of Garcia (G-S-94), and OPLS-AA-2001 [78]. Simulation with these three force fields yield rather different propensities for the central alanine of AAA. Amber 94 produces the well-known preference for right-handed helical structures while the other force field yield a more balanced distribution. The authors could not reproduce the high pPII propensity for alanine with the G-S-94 force field, which can certainly be attributed to their use of an implicit solvent model. They also observed substantial differences between the Ramachandran distributions in AAA and in the alanine dipeptide in that the residue of the latter spends more time in pPII and β. These results are at variance with experimental results that show a higher pPII preference for A in AAA than in the alanine dipeptide, in qualitative agreement with Garcia’s work [79]. Again, this discrepancy points to different solvent models used by Garcia and Zaman et al.

**Figure 10:**
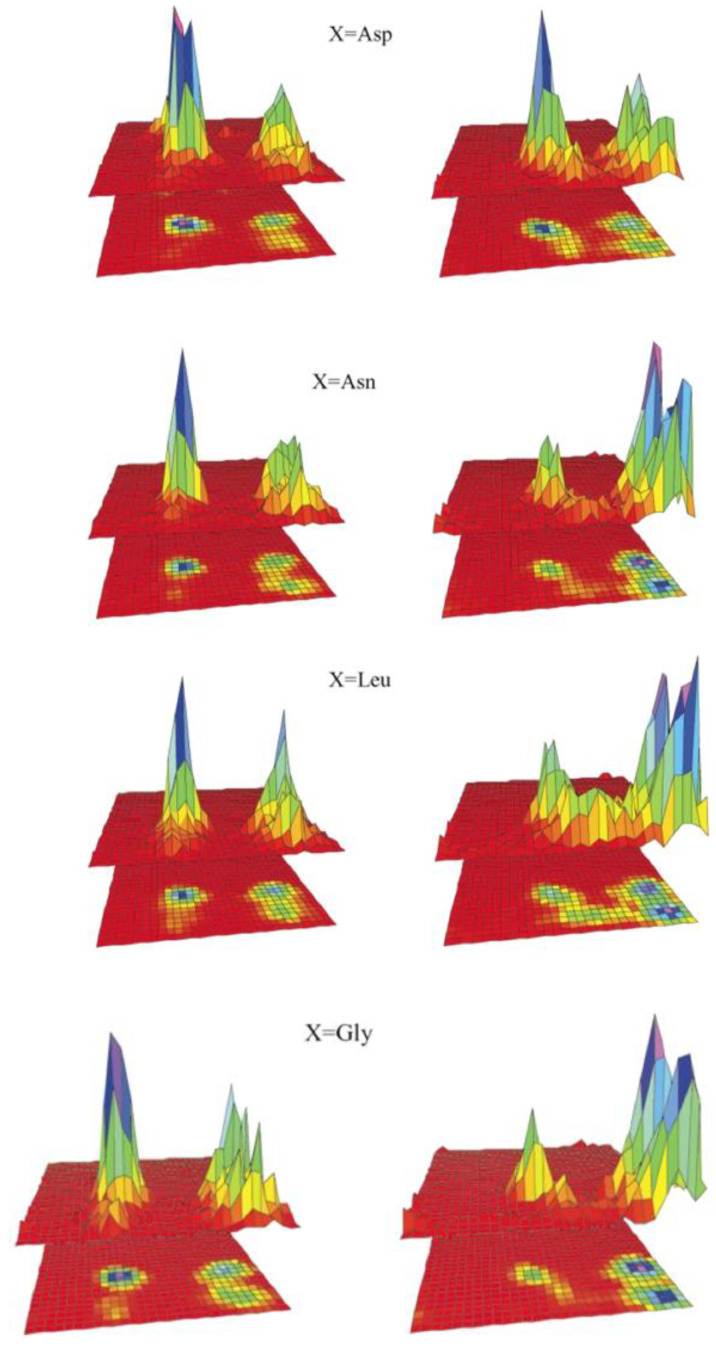
Ramachandran plots of the central alanine residues in the xAA peptides for the indicated guest residues as obtained from MD simulations with G-S-94 (**left**) and OPLS-AA-01 (**right**) force fields. Details are described in the text. Taken from [78] and modified.

Figure 10 displays the propensities for four different representative neighbors obtained with the G-S-94 and OPLS-AA-2001 force field. The predicted changes are considerable but very much force-field dependent. For GAA, which one could use as a reference system, the G-S-94 force field produces a Ramachandran plot for the central alanine that is dominated by right-handed helical conformations. On the contrary, the OPLS force field produces a dominance of pPII and β-strand. With G-S-94, replacing G by L, N or D keeps the high helical propensity while causing some redistribution between pPII and β-strand. The OPLS force field yields an increased sampling of the right-handed helical and bridge region for G→L and an overall increase in the helical population for G→D. The distributions obtained with Amber 94 are not indicative of massive NNI influence, as for all the guest residues the α-helix population is dominant. There is no doubt that the results of these calculations are important in that they suggest that NNIs can produce substantial changes in the Gibbs energy landscape of residues and their conformational entropy. However, without experimental validation, it is problematic to employ the obtained population changes for a quantitative assessment of the influence of NNIs on the energetics of unfolded polypeptides and proteins.

## 5. Experimental Results

### 5.1. NMR on Denatured Proteins

As indicated above, the first experimental results indicating that NNIs affect the structural distributions of denatured proteins came from NMR studies. They rely to a significant extent on the use of a J-coupling constant, which reflect the degree of through-bond interaction between two nuclear spins, which are generally one, two or three bonds apart. Their general dependence on dihedral backbone angles is described by Equation (6) (*vide supra*).

Penkett et al. used ^3^J(H^N^H^Cα^) coupling constants of a denatured fibronectin binding protein to conclude that β-branched and aromatic neighbors shift these values up [61]. The authors interpreted this observation as indicating a shift to more negative average *φ*-values of the respective conformational ensemble. An even more thorough study was conducted by Peti et al., who analyzed ^3^J(H^N^H^Cα^) coupling and chemical shifts of three denatured proteins, namely, ubiquitin, disulfide reduced, carboxymethylated lysozyme and a so-called all-A-α-lactalbumin (all-A means that all cysteines were replaced by alanines) [80]. ^1^H,^15^N-HSQC spectra were interpreted as indicative of a random coil conformation in which right-handed helical and pPII/β-regions are populated. This notion was further supported by their observation that the average ^15^N chemical shifts of the amino acid residues (taken over all residue of a given type in the investigated proteins) correlate with the corresponding chemical shifts derived from the (restricted) coil library of Smith et al. [81]. However, if these proteins were really sampling a random coil type ensemble there should be no NNIs of significance. This, however, does not seem to be the case. Peti et al. showed that the nearest neighbor-induced chemical shift changes reported by Braun et al. [82], based on ^15^N measurements of unblocked GGxA peptides (x represents all natural amino acid residues), correlate with the nearest neighbor effects on leucine residues in the set of unfolded proteins [83]. They attributed these changes to conformational changes. Correlations between ^15^N chemical shift and ^3^J(H^N^H^Cα^) coupling constant changes support this notion. These results seem to confirm the observation of Penkett et al., that branched and aromatic neighbors produce more negative *φ*-values [61]. If Peti et al. interpreted the neighbor dependence of the ^15^N chemical shifts correctly, their results invalidate the isolated pair hypothesis, which implies that the conformational ensemble of the investigated proteins cannot be an ideal random coil.

### 5.2. Structural Analysis of Homopeptides

The above NMR based analyses rely very much on averages over many amino acid residues in the considered denatured proteins and in the utilized coil libraries. We wonder whether such a procedure could obfuscate information about the conformational propensities of residues and their dependence on nearest neighbors. Moreover, averaging over different ensembles might lead to very similar coupling constants, so that changes in the latter are difficult to interpret, particularly if one relies only on a single type of coupling parameter. An alternative approach in this regard utilizes short peptides, which, owing to their limited length, cannot adopt any regular secondary structure. For a long period of time, blocked dipeptides were considered suitable model systems to explore intrinsic conformational propensities of amino acid residues. Ramachandran and Flory used them to explore the sterically allowed region of the Ramachandran plot [3,69]. The alanine dipeptide has been the system of choice for multiple MD simulations [84,85,86,87,88,89]. More recently, they have been used to experimentally determine conformational preferences in water and related blocked tripeptides even for the investigation of nearest neighbor interactions [17,23,90,91,92,93,94]. The preference for blocked dipeptides over, e.g., unblocked tripeptides, was generally based on the assumption that the charged terminal groups of, e.g., tripeptides, could affect conformational propensities [95,96]. However, experimental evidence reported by Toal et al. revealed that this is not the case for trialanine (A_3_) and trivaline (V_3_) [79]. Kallenbach and coworkers chose AcG_2_xG_2_NH_2_ host–guest peptides [97,98]. Our research group has embarked on a thorough investigation of unblocked tri-, tetra-, and pentapeptides to determine the intrinsic conformational propensities and NNI effects [24,42,43,44,45,99,100,101]. Contrary to blocked dipeptides, this choice provides some more natural context to the investigated amino acid residue. In what follows, we will review these works with an emphasis on NNIs.

We start this discussion with a focus on alanine. The respective amino acid residue has long served as model system for the exploration of the Ramachandran space. The Ramachandran plot for the alanine dipeptide that solely reflects steric exclusion and electrostatic interactions looks very much like Figure 1. Thus, it fully represents the local aspect of an ideal random coil behavior. Very similar plots were obtained with more sophisticated molecular dynamics simulations in explicit water [91,92,102,103]. Hence, it came as a surprise when Shi et al., based on ^1^H NMR and UVCD data for a hepta-alanine peptide (XAO-peptide, Ac-X_2_A_7_O_2_-NH_2_, X represents aminobutyric acid) in water, arrived at the conclusion that the peptide predominantly samples a basin assignable to the pPII conformation [104]. Up to this point this conformation had been associated with the *trans* conformation of proline in oligo- and poly-proline peptides, even though some early UVCD studies of Tiffany and Krimm had indicated that poly-L-lysine and poly-L-glutamic acid could adopt this conformation [105]. Their results were later corroborated by vibrational circular dichroism studies [106].

Since there was no obvious reason for alanine to prefer pPII, the results of Shi et al. became highly controversial in the field. Scheraga, Liwo and coworkers re-analyzed the data of Shi et al. based on the results of MD simulations and arrived at the conclusion that there is no specific preference of alanine for pPII [102,103,107]. Small-angle X-ray scattering data were found to be inconsistent with a conformational ensemble dominated by pPII [103]. This discussion overlooked the fact that early coil library studies had already indicated the very high pPII propensity of alanine (*vide supra*), thus lending credibility to the results of Shi et al. [40].

After the paper of Shi et al. was published, their results were sometimes interpreted as indicating that the alanine sequences could adopt a stable pPII-helix [108,109,110]. Some wording chosen by the authors certainly facilitated this reading, but in a follow-up paper they actually found no evidence for any cooperative nearest neighbor interactions between alanines in GGAAGG and GGAAAGG peptides [98], which would be needed for the formation of a stable pPII helix. Nevertheless, their work triggered a discussion of the so-called reconciliation problem, namely, the apparent contradiction between the occurrence of pPII helices in denatured proteins and their well-established behavior as a self-avoiding random coil [30,74].

Spectroscopic studies on alanine-based oligopeptides suggest that some cooperativity between the pPII states of alanine residues actually exists, in line with the MD results of Garcia (*vide supra*). At an early stage of the debate about the alleged pPII propensity of alanine, Schweitzer-Stenner et al. combined IR, polarized Raman and vibrational circular dichroism (VCD) data to show that the unblocked tetra-alanine A_4_ has a higher pPII propensity than tri-alanine (A_3_) [111]. This result was corroborated by a Raman optical activity study of McColl et al. [112]. However, both studies were qualitative in nature in that they did not report any numbers reflecting conformational propensities. This gap was filled later by more quantitative studies that utilized NMR J-coupling constants in addition to the amide I’ band profiles in IR, Raman and VCD spectra. The obtained results suggest that the central alanine residue in A_3_ has a slightly higher pPII propensity than the one in GAG, namely, 0.84–0.9 for the former and 0.72–0.8 for the latter [76,84,113]. This difference looks small, but it is indicative of a Gibbs free energy change of ca. 3 kJ/mol in favor of pPII (for A_3_).

Graf et al. measured the ^3^J(H^N^H^Cα^) and ^2^J(NC_α_) coupling constants for a series of A_n_ peptides (*n* = 3–7) [114]. While the former reflects average *φ*-values, the latter depends on the *ψ*-value of the residue that precedes the utilized amide nitrogen. The authors used the measured coupling constants in constrained MD simulations from which they obtained a slight stabilization of β-strand over pPII with increasing residue number. However, the obtained changes might be within the error limits of their analysis. Two other spectroscopic studies suggest that an increasing length of alanine sequences stabilizes non-extended structures over pPII, in line with predictions [75]. Verbaro et al. combined vibrational spectroscopy and fluorescence energy transfer experiments with the coupling constants of Graf et al., to show that the individual alanine residues of the unblocked A_5_W peptide exhibit pPII fractions between 0.65 and 0.75 [115]. These values are lower than the those observed for trialanine, but they are still way higher than any predictions obtained with steric exclusion and MD calculations. The slight destabilization of pPII benefits right-handed helical conformations for residues 2–4, in line with predictions of Gnanakaran and Garcia. The fifth alanine residue behaves differently in that it exhibits a more pronounced β-strand population. The latter could reflect interactions with the aromatic W-residue at the C-terminal, which exhibits a mixture of pPII and β-strand.

Shi et al. had initiated the debate about the pPII propensity of alanine with a spectroscopic analysis of the XAO peptide (*vide supra*). They deduced a rather high propensity (mole fraction) value of ~0.9 from their data. While later studies on shorter peptides yielded similar values, it is higher than the values Verbaro et al. reported for the alanine residues in A_5_W. Moreover, a conformational ensemble of XAO totally dominated by pPII sampling would be inconsistent with the radius of gyration obtained from the SAXS experiments [103]. A more realistic picture emerged from the study of Schweitzer-Stenner and Measey [116], who combined IR, Raman and VCD band profiles with the ^3^J(H^N^H^Cα^) values of Shi et al. with the results of MD simulations of Scheraga, Liwo and collaborators [116]. The authors obtained pPII propensities in the 0.55–0.7 range for the three central alanine residues of the peptide, while the values are much lower for the residue in proximity of X and O. They were found to sample an exceptional large number of different turn-supporting structures and to exhibit larger β-strand propensities than alanine in short peptides. The results of this study are in line with those of Verbaro et al. [115], but they additionally suggest that alanine can be heavily influenced by the conformational distribution of neighbors. This was later confirmed by Toal et al. [99] (*vide infra*). Regarding the debate about the pPII propensity of alanine, the results of Schweitzer-Stenner and Measey hit a middle ground between two extreme views, namely, that of a nearly all pPII XAO peptide [104] and the results of MD simulations and short-angle X-ray studies that suggested that there is no exceptional pPII propensity of alanine at all [103,107].

**Figure 11:**
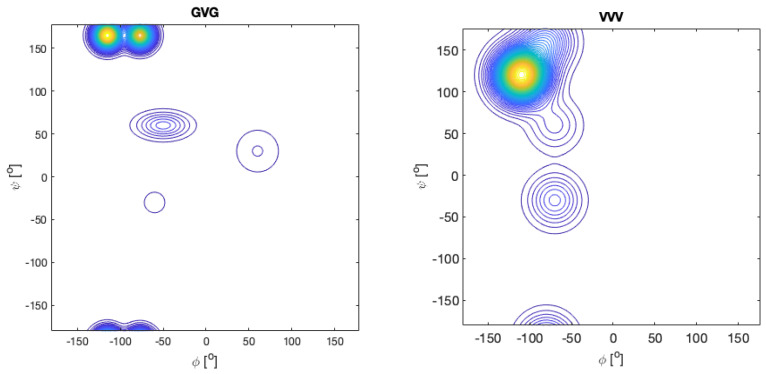
Ramachandran plots of the central valine residue of GVG (**left**) and VVV (**right**). The plots were produced using the Gaussian model of Schweitzer-Stenner et al. with the parameters reported for these peptides [42,72].

We now move to a discussion of other homopeptide sequences. Early work of Eker et al. suggested that the central valine in unblocked V_3_ has a very high β-propensity [117,118]. The latter was later confirmed by Graf et al. who reported a value of 0.52 for β, 0.19 for pPII and 0.29 for right-handed helical conformations [114]. Schweitzer-Stenner, who combined the J-coupling values of Graf et al. with amide I profiles, reported an even higher β-strand value (0.68), which comes at the expense of pPII and right-handed helical sampling (0.16 for both) [72]. These values seem to be more in line with the absence of a significant signal in the UVCD spectrum of V_3_ [118]. They should be compared with the conformational distributions of valine in GVG, for which Hagarman et al. reported a β-strand propensity of just 0.38 [42]. The remaining population of V is distributed over pPII and several turn-forming conformations, including γ-turn. The Ramanchandran plots of GVG and VVV are shown in Figure 11. They illustrate the large influence that the two terminal valine residues have on the central valine residue, which involves changes in the population and basin position. Taken together, these results are indicative of strong NNIs that cause a predominance of β-strands, which, with respect to pPII, is stabilized by ca. 3.7 kJ/mol in VVV.

Results obtained for other homo-tripeptides and related GxG peptides are noteworthy. For GKG, Hagarman et al. reported a rather balanced distribution in the upper left panel of the Ramachandran plot (mole fractions of 0.5 and 0.41 for pPII and β-strand, respectively) [42]. The distribution of the central residue of KKK reported by Verbaro et al. is significantly different [113]. The usual two basin distribution comprising pPII and β is merged into one broad basin centered at *φ*,*ψ* = −95°, 170°. The authors termed this a distorted pPII conformation. This notion seems to be justified by a UVCD spectrum that is still very much pPII like, though with a more symmetric couplet. The result is at least qualitatively consistent with earlier findings for poly-L-lysine and an hepta-lysine peptide [105,119]. Apparently, the changes caused by NNIs in this peptide are quantitative and qualitative in that they can change the populations as well as the positions of the basins.

Another GxG-x_3_ comparison has been carried out for aspartic acid. The Ramachandran of GDG is rather peculiar. The pPII population is low (0.2), whereas the β-strand is comparatively highly populated (0.48). In addition, aspartic acid was found to sample type II’ β-turn-supporting conformations and to a significant degree asx-turn conformations, which lie in the upper right quadrant of the Ramachandran plot (Figure 11) [43,45,59]. The latter do not appear in coil library Ramachandran plots for these aspartic acid residues, but they occur frequently in proteins [60]. In fully protonated DDD, the β-strand population of the central residue is nearly identical with the one of GDG, but the shape of the distribution was found to be different [120]. However, NNIs populate right-handed helical (3_10_)-supporting conformations at the expense of pPII and asx. The former lies slightly below the type II′ β-turn-supporting conformation populated in GDG [45]. Interestingly, upon deprotonation of the D-residues, the distribution looks very much like the one observed for KKK, but with a less negative (more pPII-like) *φ*-angle. A similar result was obtained for ionized GDG by Rybka et al., though with more separated pPII and β-basins [45].

**Figure 12:**
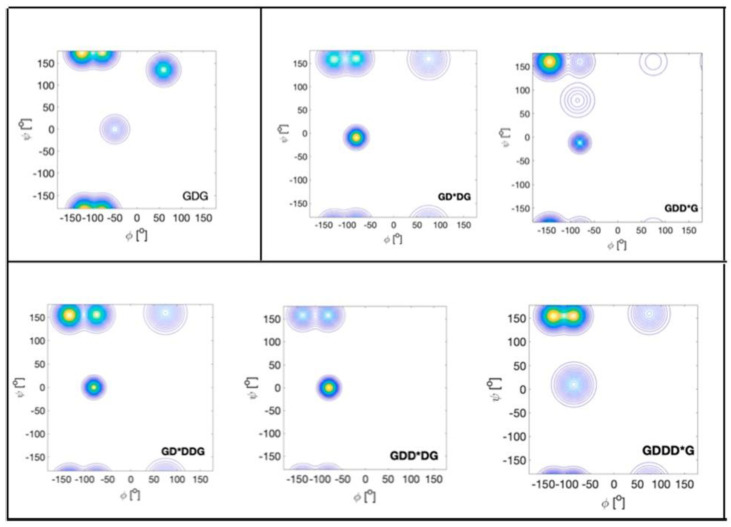
Ramachandran plots of non-terminal (D) residues of the indicated fully protonated oligo-L-aspartic acid peptides. Figure reproduced from [101] with permission, 2021, American Chemical Society.

Recently, our research group has explored the conformational landscape of unblocked GxxG and GxxxG peptides. Contrary to the above tripeptides, the presence of terminal glycine residues allowed us to determine the Ramachandran plots of all x-residues. Here, we start with x = D (protonated). The Ramachandran plots are shown in Figure 12. There are numerous noteworthy observations. First, the corresponding peptide Ramachandran plots are rather different, which already indicates that the NNIs are operative. For all D-residues, we obtained a comparatively high population of type I/II′ (i + 2)-turn-forming structures. The asx-turn basin is still populated for residue D1 of GDDG and for D1 and D3 of GDDDG. pPII and β-strand populations are comparable, with the exception of the second D residue of GDDG, where β-strand is even more populated than it is in GDG. An investigation into the aspartic acid dipeptide at acidic and neutral pH led to the conclusion that the above differences between ionized and protonated DDD reflect interactions between the terminal carboxylate group and the aspartate side chains, which are naturally absent in denatured proteins. Hence, it is not surprising that, e.g., the coil library distribution of aspartic acid in DDD segments resembles more the one observed for protonated DDD [120]. The results obtained with fully protonated D-containing peptides should be considered as representing the properties of D-containing homo-segments in numerous disordered segments or proteins. A detailed discussion can be found in Milorey et al. [101].

What do the results of the studies on the above homopeptide sequences have in common? For the D- and R-sequences, NNIs seem to produce a distribution with a nearly equipartition between pPII and β. The respective mole fractions of, e.g., GRRG and GRRRG, suggest that while the N-terminal R resembles to some extent the central residue of GRG (i.e., pPII dominance over β), the distributions of the other R-residues are much more balanced [100]. A similar equipartition effect seems to be operative in trilysine, where it is accompanied by a merger of pPII and β-basins. For D, this conformational balancing appears only in GDDDG. NNIs cause a reorganization of the population of turn-forming peptides for both R and D. The central R residue of GRRRG features some measurable populations of right-handed helical conformations, while the central residue of GDDDG exhibits an increased population of type II’/I β-turn-forming structures. A Hellinger distance analysis of Milorey et al. suggests that the distributions of the tetra- and pentapeptides are distinct from that of the respective GxG, while they show some similarity with respect to each other [100,101]. This analysis reveals that the basin positions in the Ramachandran plots of the investigated GxxG and GxxxG peptides are not very different. For trivaline and oligo-alanines, the NNIs seem to stabilize the already dominant conformer, but the length-dependent propensity of alanine for right-handed helices leads to stabilization of the helical conformations [27,75].

Recently, Schweitzer-Stenner et al. used NMR spectroscopy to investigate the cationic state of GKKG. They found that the downstream lysine residue is more effected by NNIs than the upstream one. The Ramachandran for the former looks very much like the one obtained for KKK, but with pPII and β-strand bassines clearly separated [121].

### 5.3. Structural Analysis of Heteropeptides

**Figure 13:**
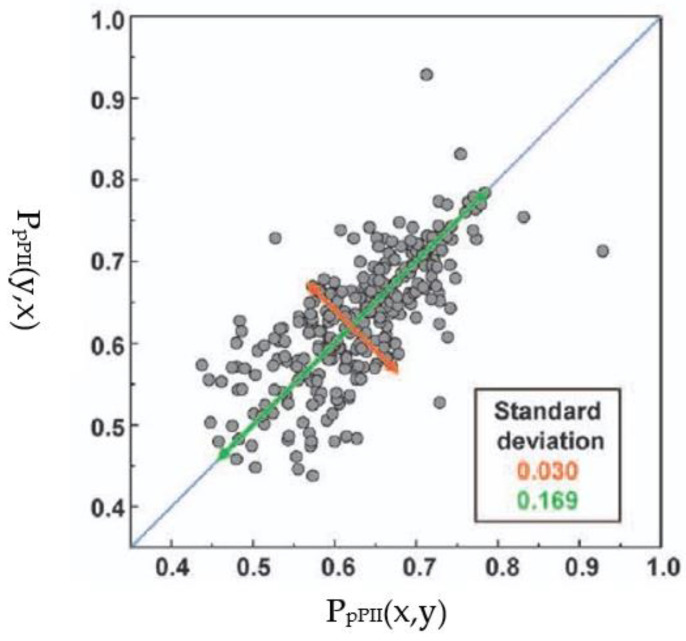
Correlation plot of the pPII propensities of amino acid residues in blocked tripeptides with the residue sequence xy versus the respective pPII propensities of the corresponding yx peptides. Proline residues were excluded. The figure was taken from [122] and modified.

In this paragraph we discuss two experimental approaches aimed at exploring NNIs in short peptides. We start with the work of Cho and colleagues. Oh et al. used UV CD and ^3^J(H^N^H^Cα^)-values of blocked tripeptides to assess the nearest neighbor interactions between all 20 natural amino acid residues (the authors used the term dipeptides, but that is not in line with literature terminology for blocked peptides) [93,122]. Since they did not assign individual amide proton signals, they used an average of the two ^3^J(H^N^H^Cα^) coupling values. They compared the average pPII population of the corresponding xy and yx pairs of the residues, which they deduced from the average ^3^J(H^N^H^Cα^) coupling by applying a simple two-state (pPII-β strand) model. The large aspect ratio of the standard deviation along the diagonal (0.169) and the antidiagonal (0.03) in Figure 13 was interpreted as indicating that the effective propensities of the corresponding xy and yx pairs are very similar. In other words, the amino acid residue type rather than the sequence matters.

**Figure 14:**
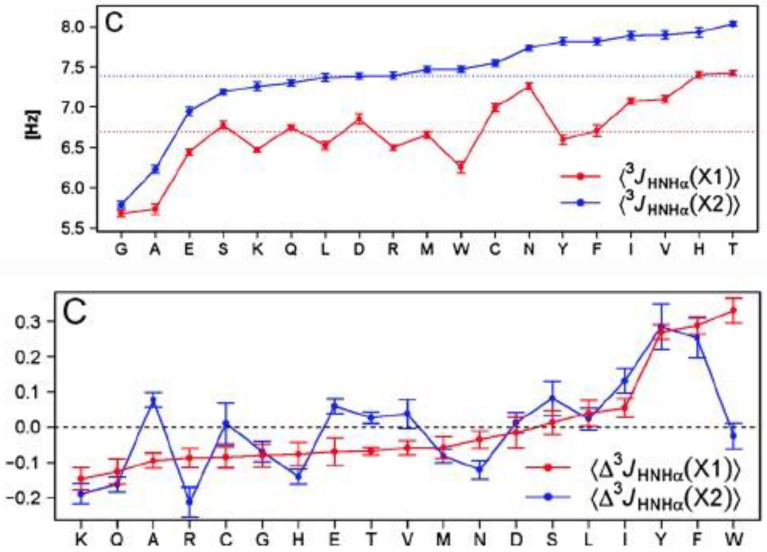
Upper panel: Average J-coupling constants of the indicated amino acid residues in blocked xy-tripeptides. Lower panel: Average effect of the upstream and downstream neighbors on the ^3^J(H^H^H^Cα^) constant of the indicated residues of blocked xy-tripepetides. Note that the authors’ x_1_ and x_2_ correspond to x and y of the notation used in this article. The character C in the upper left corner indicates that these figures are part of a figure set in [94], from where they were taken and modified.

In a follow-up paper, Jung et al. investigated the same large set of peptides, but this time they assigned chemical shifts and thus J-coupling constants to the N- and C-terminal residues [94]. Here we focus on their ^3^J(H^N^H^Cα^) data because this parameter has a clearly established structure dependence. Figure 14 (upper panel) shows the average ^3^J(H^N^H^Cα^) of their x and y residues for the 19 investigated amino acid residues (proline omitted). The averaging was done over all neighbors. Several aspects of the plots are noteworthy. Just based on their ^3^J(H^N^H^Cα^) values there seem to be three classes of residues. The first one contains residues with values significantly below the respective average (6.7 Hz for x and 7.4 Hz for y). It solely contains alanine and glycine. The second one contains values significantly above the averages. Its members are N, I, V, H and T for x and N, Y, F, I, V, H and T for y. If changes of ^3^J(H^N^H^Cα^) would solely reflect pPII/β-ratios, class 1 residues would have a high pPII propensity, while class 2 members would prefer β-strand. The remaining residues (class 3) fluctuate around the respective average values. The second observation is that the standard deviations of the average values are small. This seems to suggest that the nearest neighbor dependence of ^3^J(H^N^H^Cα^) is weak. Third, the averages over all J-coupling constants are different (larger for y than for x). This seems to reflect some end effects, which is astonishing because one of the constantly stated arguments for the use of blocked peptides is the proposed absence of end effects.

The plots in the lower panel of Figure 14 convey a slightly different message. It depicts the average change of ^3^J(H^N^H^Cα^) caused by the respective residues that constitute the abscissa. The data suggest that if positioned at x most of the residues exert nearly the same rather moderate influence, with the aromatic residues Y, F and W as exceptions. The latter all increase the ^3^J(H^N^H^Cα^) values of their neighbors. More variations were observed for the y position, where K, R, Y, H and N cause a reduction in their neighbors ^3^J(H^N^H^Cα^), while Y, F and W at position x again cause a substantial increase in this coupling constant.

**Figure 15:**
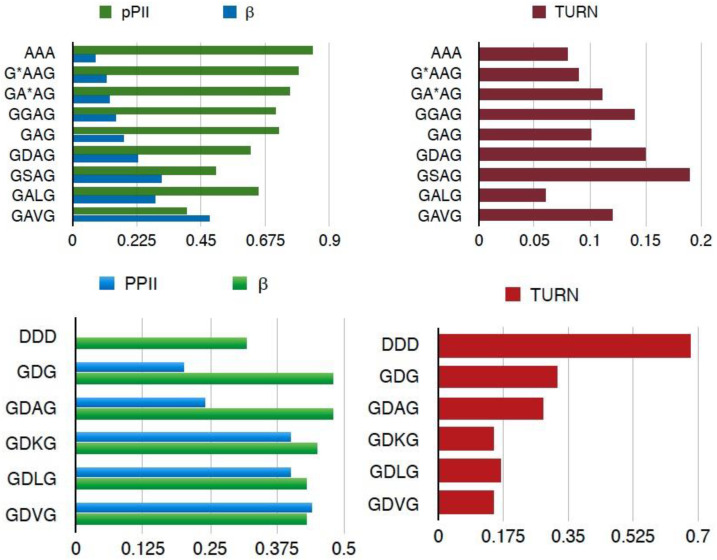
Mole fractions of pPII, β-strand and turn-forming conformation of alanine (**upper panel**) and aspartic acid (**lower panel**) in the indicated tri- and tetrapeptides. The turn fraction was calculated as the sum over the occupation of all non-pPII/β-strand conformations. Note, that the color codes for the two panels are different. Taken from Toal [126].

**Figure 16:**
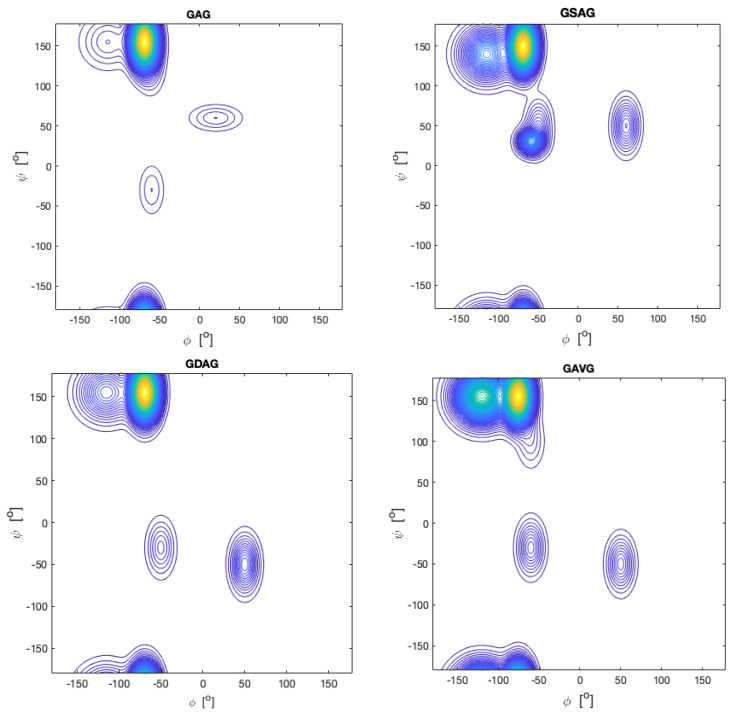
Ramachandran plots of alanine residues in the indicates peptides. Produced with data reported in [99].

Now, we turn to the work on unblocked peptides carried out in our research group and in the Schwalbe laboratory in Frankfurt. Toal et al. conducted a large number of investigations into GxyG-type tetrapeptides by using vibrational and NMR spectroscopy. Here, we focus on the most important aspects of their results. Figure 15 shows the mole fraction of a series of GxAG, GAyG, GxDG and GDyG peptides. The Ramachandran plots for the alanine-containing series are depicted in Figure 16. In all these cases, the influence of the x- and y-neighbors is obvious. For alanine they reduce the pPII fraction quite substantially. The effect is most pronounced for valine. In the case of aspartic acid, the nearest neighbors increase the pPII propensity of D at the expense to turn conformations. On both cases, NNIs move the system towards equipartition, though to a different degree.

Toal et al. found that propensity-related NNI effects are much less pronounced for pairs of lysine, leucine and valine [99]. Interestingly, however, NNIs were found to cause, in part, substantial changes in the basin coordinates. These results thus underscore the notion that changes in individual coupling constants can be very misleading in that they could reflect changes in populations and basin coordinates. Only the use of multiple coupling constants and vibrational spectroscopy data lead to a meaningful result.

**Figure 17:**
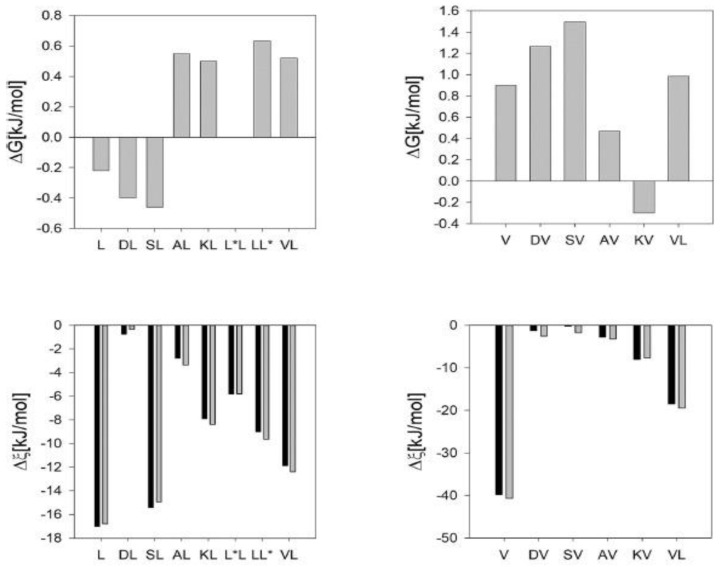
Plots of the Gibbs free energy, enthalpy and entropic contribution to the pPII-β strand equilibrium of aspartic acid, serine (**upper panel**), leucine and valine (**lower panel**) in the presence of indicated neighbors in GxyG tetrapeptides. In the lower panel the character ξ represents ΔH (black) and TΔS (grey). These figure merges two different figures from [99].

In addition to exploring the conformational distributions of GxyG peptides at room temperature, Toal et al. measured the temperature dependence of the ^3^J(H^N^H^Cα^) constants. Earlier ^1^H NMR experiments on GxG peptides had shown that these data sets can be analyzed rather accurately in terms of a two-state model that describes the equilibrium between pPII and β-strand [45]. This yields very informative values for ΔH and TΔS. Figure 17 compares the thermodynamic parameters of L and V residues in the presence of different neighbors. For L the ΔG-values of pPII/β-strand equilibria at room temperature vary between 0.5 and −0.5 kJ/mol (~0.2*RT), in line with the reported very limited influence of NNIs on L at room temperature. However, except for GDLG, the corresponding enthalpic and entropic differences of the investigated GxLG and GLyG peptides are considerable. This notion particularly applies to GSLG and GVLG. For V-containing tetrapeptides, the thermodynamic parameters convey a different message. While ΔH and TΔS values are large for GVG, corresponding values of V-containing tetrapeptides are small, with the notable exception of GVLG. If both enthalpic and entropic values are high, then the entropy will win at temperatures at which proteins thermally unfold. This leads to a stabilization of β-strand conformations. The rather small Gibbs energy differences between pPII and β-strand at room temperature obtained for all residue pairs, depicted in Figure 17, are due to enthalpy–entropy compensation and the close proximity of the isoequilibrium point to room temperature. Toal et al. showed that the Gibbs energy difference of a rather large number of GxG peptides (x = L, V, I, S, K, Y, W and F) become practically identical at a temperature of 302 K. The isoequilibrium of another group (x = E, R, M and N) was observed at 312 K [24]. The occurrence of isoequilibria points can be related to an enthalpy–entropy compensation and a common origin of enthalpic and entropic differences, namely, solute–solvent interactions [123,124,125], which in the case of GxyG peptides can obscure NNI effects. What this implies for our understanding of thermal unfolding of proteins has still to be understood.

In a follow-up study, Schweitzer-Stenner and Toal compared Ramachandran plots of GxG and GxyG by means of Hellinger distance calculations [73]. We reiterate that Hellinger distances are more sensitive to changes in basin coordinates than to redistributions of populations. Their values suggest that, e.g., distributions of GAG are only modestly dissimilar from those of the alanine residues in the above tetrapeptides. Compared with GDG, however, K, L and V as neighbors produce very dissimilar distributions of D. Interestingly, a significant dissimilarity was also obtained for valine if flanked by D, S and L, which predominantly reflects basin rather than population changes. Moreover, the authors calculated the Hellinger distances for corresponding pairs in the coil library of Ting et al. They obtained much lower values, which suggests that for the investigated residue pairs the averaging over either the upstream or downstream residues obfuscates to some extent the NNIs between residues.

While the experimental data presented in this and preceding paragraphs reveal substantial NNI effects between amino acid residues in short peptides and in coil libraries they do not *per se* imply a violation of the isolated pair hypothesis. In principle, it is thinkable that just the different nature of neighbors causes the observed changes in basin population and coordinates. A breakdown of the isolate pair hypothesis requires that the population of a conformation of a residue depends on the conformation adopted by its neighbors. Recently, Schweitzer-Stenner and Toal showed that the available GxyG data set is diagnostic of an anticooperative pPII-β-strand interaction where the pPII of one residue stabilizes the β-strand of neighbors, thus destabilizing the respective pPII conformation [127]. For some residue pairs, this interaction becomes significant only at high protein-melting temperatures, because of the large enthalpic and entropic differences between pPII and β-strand (*vide infra*). The authors utilized the derived temperature dependence of the NNIs between K and V in GKVG to predict the temperature dependence of the UVCD spectrum of the unfolded Max3 peptide (VK)_4_V^D^PPTKKV(KV)_2_.

The work of Toal et al. as well as the homopeptide studies discussed in the preceding paragraph revealed the necessity to use a set of J-coupling constants in conjunction with vibrational spectroscopy data to arrive at a quantitative assessment of NNI. Just probing ^3^J(H^N^H^Cα^) and its changes in the presence of different neighbors does not allow for a valid structural analysis. In order to substantiate this notion, a closer look at J-coupling constants is helpful. Let us start with alanine. If one averages over all alanine neighbors the obtained value for ^3^J(H^N^H^Cα^) is 6.36 Hz and carries a standard deviation of 0.4 Hz, which might point to moderate NNIs (data were taken from Toal et al. [99]). For ^3^J(H^N^C′) the average value is 1.23 Hz with a standard deviation of 0.08 Hz. These values seem to suggest weak NNIs. However, a closer look informs that these averages have very limited meaning. Valine as a neighbor increases ^3^J(H^N^H^Cα^) from 6.1 to 6.6 Hz, which is a significant increase. Concomitantly, ^3^J(H^N^C′) increases from 1.18 to 1.27 Hz. These correlated changes combined with an even more significant increase in ^3^J(H^Cα^C′) from 1.02 to 2.56 Hz reflect a drastic decrease in the pPII propensity from 0.8 to 0.38. Again, if one would be only concerned about the average ^3^J(H^Cα^C′) value (2.15 ± 0.32 Hz), one would not expect such a significant change in the propensities by any of the investigated neighbors. The J-coupling plots for D-containing peptides underscore this conclusion. If one looks solely at the ^3^J(H^N^H^Cα^) values, a standard deviation of 0.47 Hz (relative value = 0.06) for an average of 7.42 Hz suggest that the nearest neighbor-induced changes are significantly smaller than, e.g., the differences between the average values obtained for A and D. However, respective ^3^J(H^N^C′) and ^3^JH^NCα^C′) variations are way more pronounced, thus indicating significant NNI-induced changes, in line with the analysis of Toal et al. [99]. For the series of arginine homopeptides investigated by Milorey et al., the standard deviation for ^3^J(H^N^H^Cα^) is very small (the relative value is just 0.04) [100], which would lead to the conclusion that the mutual influence of arginines on each other is weak. However, a look at the large standard deviation of the average ^3^J(H^N^C′) reveals that such a conclusion would be incorrect. It should be noted in this context that the changes in the VCD strength of the excitonically coupled amide I′ modes of GxyG peptides, as reported by Toal et al., are further indicators of substantial NNI-induced structural changes [99].

Taken together, the studies described in this paragraph corroborate the occurrence of residue specific NNIs. The observed correlation between the pPII and β-strand propensities of the neighbors indicate that NNIs are conformation dependent, which implies a breakdown of the isolated pair hypothesis.

## 6. Summary and Outlook

Nearest neighbor interactions between amino acid residues in unfolded and denatured proteins started to attract attention nearly 25 years ago. Their relevance stems from the fact that their occurrence could indicate a violation of the isolated pair hypothesis of Flory, on which his random coil model for unfolded polypeptides and proteins was built. At the beginning information about nearest neighbor interactions came from coil library analyses, MD simulations and, to a limited extent, from NMR data on denatured proteins. The results of coil library analyses provided the clearest evidence for a substantial influence of NNIs on conformational propensities of amino acid residues in coil-like structures. Only recently has experiments on model peptides provided a more quantitative assessment of NNI in terms of propensity changes and thermodynamic interaction parameters. Unfortunately, a unifying picture did not yet emerge from the available data. Early coil library and NMR data indicate that the upstream presence of aliphatic and aromatic residues might cause a conformational redistribution to more extended structures. The extensive set of coil library-based Ramachandran plots of Ting et al. suggest that NNI mostly affect the population of the basin associated with right-handed helical conformations. The few MD-simulation studies aimed at exploring NNI effects reveal that the emerging results depend on the choice of the force fields. Experimental studies on tetra- and a few pentapeptides reveal a complicated picture in that they show that NNI effects are highly residue and sequence dependent. While they mostly cause redistributions between pPII and β-strand conformations, modest populations of turn-forming conformations occur in homopeptide sequences.

In view of the complexity indicated above one might wonder whether studying NNIs is worth the effort. While there has been considerable initial interest in the subject over a period of ca. 15 years after the emergence of first evidence for detectable NNIs in denatured proteins, it seems that it has been put on the backburner, particularly by the computational community. Recent force-field developments were guided by conformational analyses on block dipeptides, which thus explicitly excludes NNIs [128]. Conformational studies on poly-peptide sequences with different net charges were predominantly aimed at exploring the classical polymer physics parameters, such as the radius of gyration, average interresidue distance and scaling factor, rather than on conformational propensities and nearest neighbor interactions. We think that an explicit consideration of NNIs is necessary for a variety of reasons. First, even though our experimental studies suggest that NNIs frequently lead to a randomization of individual Ramachandran distributions and correlation effects between pPII and β-strand conformations, they cause a reduction in conformational entropies. This notion is in line with computational studies on unfolded proteins [18,77]. In view of the relevance of the conformational entropy for protein–protein and protein–DNA interactions associated with disorder->order or order->disorder transitions, a correct assessment of the conformational entropies of the involved IDPs or disordered segments seems to be pivotal. Second, since solvation plays a major role in determining conformational propensities and NNIs, the notion that the solvation Gibbs energy of an unfolded protein is just the sum of the residue solvation energies can no longer be maintained [129]. Third, given the relevance of MD simulations for the study of IDPs and their biological functions, the utilized force field should be able to account for the influence of NNIs. In view of the fact that most of the current force fields cannot even properly reproduce experimental data of model peptides [25,26,130], the field is not even close to achieving this goal. Fourth, it is very likely that residual structures of protein segments are relevant for the initial phase of protein folding and peptide/protein self-assembly [131,132,133].

When it comes to the study of NNIs one might wonder whether a further exploration of coil libraries or studies of short peptides can fill the void. The advantage of the former is the large data sets; its disadvantage is the fact that coil distributions might still not be representative of residues in unfolded proteins, because the protein context and different degrees of solvent accessibility cannot be ignored [45]. The latter issues are addressed with studies on short model peptides, but performing an extensive analysis, such as the one by Toal et al., for all combinations of amino acid residues is out of question. Moreover, one has to take into account that studies on short peptides may not provide the full picture, even for longer polypeptides that are incapable of folding. The reason for this deficiency is that cooperative effects that support the population of right-handed helical structures are difficult to obtain from an analysis of tetra- and pentapeptides. According to the Zimm–Bragg or Lifson–Roig model, cooperative NNIs between residues in helical conformations can become more relevant with increasing length of the oligo/poly-peptide [134,135]. This could actually explain the observation that coil library distributions indicate more right-handed helical content than the Ramachandrans of short peptides [44]. To maneuver in this rather complex landscape, a reduction strategy is called for that would identify the NNIs between a limited number of residues, representing groups with aromatic, polar, charged and aliphatic side chains. Possible candidates are F, S, R and V. One might add L to the aliphatic group since β- and γ-branched aliphatic residues behave differently [99]. 

Available data sets for A, L and V are already considerable but might need to be complemented by studies on pentapeptides, since the influences of up- and downstream neighbors are apparently not additive. D plays a special role because its side chain can interact with the backbone. Once the NNIs for a complete set of sequences with these residues are determined, one could study longer peptides composed of residues for which high (Zimm–Bragg) s-parameters have been reported to elucidate the interplay between the helix and extended structures supporting NNIs. Such a data set could provide a sufficient basis for the development of a suitable MD force field and water models and substantially increase our understanding of unfolded and intrinsically disordered proteins.

## Figures and Tables

**Table 1:** List of Hellinger distances between the indicated amino acid residues independent of neighbors (left value) and in the presence of a glutamine residue at the upstream position (right value). Hellinger distance values indicating at least moderately different distributions are typed in bold. All values were taken from Ting et al. [19] and divided by 100.

	F	Y	Q	K	V	I	A	N
F	1	0.08	0.1/0.15	0.12/0.16	0.21/0.14	0.22/0.17	0.19/0.15	0.19/0.12
Y	0.08	1	0.11/0.13	0.12/0.14	0.21/0.15	0.21/0.18	0.19/0.12	0.19/0.08
Q	0.10/0.15	0.11/0.13	1	0.09	0.21/0.09	0.22/0.11	0.16/0.07	0.18/0.09
K	0.12/0.16	0.12/0.14	0.09	1	0.20/0.09	0.20/0.12	0.16/0.08	0.22/0.09
V	0.21/0.14	0.21/0.15	0.21/0.09	0.20/0.09	1	0.09/0.10	0.28/0.12	0.32/0.11
I	0.22/0.17	0.21/0.18	0.22/0.11	0.20/0.12	0.09/0.10	1	0.16/0.13	0.32/0.13
A	0.19/0.15	0.19/0.12	0.16/0.07	0.16/0.08	0.28/0.12	0.16/0.13	1	0.25/0.09
N	0.19/0.12	0.190.08	0.18/0.09	0.22/0.09	0.32/0.11	0.32/0.13	0.25/0.09	1

## References

[1] Brant D.A., Flory P.J. (1965). The Configuration of Random Polypeptide Chains. I. Experimental Results. J. Am. Chem. Soc..

[2] Flory P.J. (1953). Statistical Mechanics of Chain Molecules.

[3] Brant D.A., Flory P.J. (1965). The Configuration of Random Polypeptide Chains. II. Theory. J. Am. Chem. Soc..

[4] Uversky V.N. (2002). What Does It Mean to Be Natively Unfolded?. Eur. J. Biochem..

[5] Uversky V.N., Dunker A.K., Schweitzer-Stenner R. (2012). Why Are We Interested in the Unfolded Peptides and Proteins?. Protein Pept. Folding, Misfolding, Non-Folding.

[6] Romero P., Obradovic Z., Li X., Garner E.C., Brown C.J., Dunker A.K. (2001). Sequence Complexity of Disordered Protein. Proteins Struct. Funct. Genet..

[7] Dunker A.K., Obradovic Z. (2001). The Protein Trinity-Linking Function and Disorder. Nat. Biotechnol..

[8] Oldfield C.J., Cue B., Dunker A.K., Uversky V.N., Schweitzer-Stenner R. (2012). Binding Promiscurity of Unfolded Peptides. Protein and Peptide Folding, Misfolding and Non-Folding.

[9] Crick S.L., Pappu R.V., Schweitzer-Stenner R. (2012). Thermodynamic and Kinetic Models for Aggregation of Intrinsically Disordered Proteins. Protein and Peptide Folding, Misfolding, and Non-Folding.

[10] Vitale R.M., Andreotti G., Amodeo P., Motta A., Schweitzer-Stenner R. (2012). Structural Elements Regulating Interactions in the Early Stages of Fibrillogenesis: A Human Calcitonin Model System. Protein and Peptide Folding, Misfolding, and Non-Folding.

[11] Dobson C.M. (1999). Protein Misfolding, Evolution and Disease. Trends Biochem. Sci..

[12] Rochet J.C., Lansbury P.T. (2000). Amyloid Fibrillogenesis: Themes and Variations. Curr. Opin. Struct. Biol..

[13] Bernadó P., Bertoncini C.W., Griesinger C., Zweckstetter M., Blackledge M. (2005). Defining Long-Range Order and Local Disorder in Native α-Synuclein Using Residual Dipolar Couplings. J. Am. Chem. Soc..

[14] Schwalbe M., Ozenne V., Bibow S., Jaremko M., Jaremko L., Gajda M., Jensen M.R., Biernat J., Becker S., Mandelkow E. (2014). Predictive Atomic Resolution Descriptions of Intrinsically Disordered HTau40 and α-Synuclein in Solution from NMR and Small Angle Scattering. Structure.

[15] Swindells M.B., Macarthur M.W., Thornton J.M. (1995). Intrinsic φ, ψ Propensities of Amino Acids, Derived from the Coil Regions of Known Structures. Nat. Struct. Biol..

[16] Serrano L. (1995). Comparison between the φ Distribution of the Amino Acids in the Protein Database and NMR Data Indicates That Amino Acids Have Various φ Propensities in the Random Coil Conformation. J. Mol. Biol..

[17] Avbelj F., Grdadolnik S.G., Grdadolnik J., Baldwin R.L. (2006). Intrinsic Backbone Preferences Are Fully Present in Blocked Amino Acids. Proc. Natl. Acad. Sci. USA.

[18] Jha A.K., Colubri A., Freed K.F., Sosnick T.R. (2005). Statistical Coil Model of the Unfolded State: Resolving the Reconciliation Problem. Proc. Natl. Acad. Sci. USA.

[19] Ting D., Wang G., Shapovalov M., Mitra R., Jordan M.I., Dunbrack R.L. (2010). Neighbor-Dependent Ramachandran Probability Distributions of Amino Acids Developed from a Hierarchical Dirichlet Process Model. PLoS Comput. Biol..

[20] Shi Z., Chen K., Liu Z., Kallenbach N.R. (2006). Conformation of the Backbone in Unfolded Proteins. Chem. Rev..

[21] Schweitzer-Stenner R. (2012). Conformational Propensities and Residual Structures in Unfolded Peptides and Proteins. Mol. Biosyst..

[22] Toal S., Schweitzer-Stenner R. (2014). Local Order in the Unfolded State: Conformational Biases and Nearest Neighbor Interactions. Biomolecules.

[23] Grdadolnik J., Mohacek-Grosev V., Baldwin R.L., Avbelj F. (2011). Populations of the Three Major Backbone Conformations in 19 Amino Acid Dipeptides. Proc. Natl. Acad. Sci. USA.

[24] Toal S.E., Verbaro D.J., Schweitzer-Stenner R. (2014). Role of Enthalpy-Entropy Compensation Interactions in Determining the Conformational Propensities of Amino Acid Residues in Unfolded Peptides. J. Phys. Chem. B.

[25] Meral D., Toal S., Schweitzer-Stenner R., Urbanc B. (2015). Water-Centered Interpretation of Intrinsic PPII Propensities of Amino Acid Residues: In Vitro-Driven Molecular Dynamics Study. J. Phys. Chem. B.

[26] Zhang S., Schweitzer-Stenner R., Urbanc B. (2020). Do Molecular Dynamics Force Fields Capture Conformational Dynamics of Alanine in Water?. J. Chem. Theory Comput..

[27] Garcia A.E. (2004). Characterization of Non-Alpha Helical Conformations in Ala Peptides. Polymer.

[28] Kentsis A., Mezei M., Gindin T., Osman R. (2004). Unfolded State of Polyalanine Is a Segmented Polyproline II Helix. Proteins Struct. Funct. Genet..

[29] Richardson J.S. Ramachandran Plot. https://commons.wikimedia.org/wiki/File:Ramachandran_plot_original_outlines.jpg.

[30] Plaxco K.W., Morton C.J., Grimshaw S.B., Jones J.A., Pitkeathly M., Campbell L.D., Dobson C.M. (1997). The Effects of Guanidine Hydrochloride on the “random Coil” Conformations and NMR Chemical Shifts of the Peptide Series GGXGG. J. Biomol. NMR.

[31] Uversky V.N. (2002). Natively Unfolded Proteins: A Point Where Biology Waits for Physics. Protein Sci..

[32] Hofmann H., Soranno A., Borgia A., Gast K., Nettels D., Schuler B. (2012). Polymer Scaling Laws of Unfolded and Intrinsically Disordered Proteins Quantified with Single-Molecule Spectroscopy. Proc. Natl. Acad. Sci. USA.

[33] Müller-Späth S., Soranno A., Hirschfeld V., Hofmann H., Rüegger S., Reymond L., Nettels D., Schuler B. (2010). Charge Interactions Can Dominate the Dimensions of Intrinsically Disordered Proteins. Proc. Natl. Acad. Sci. USA.

[34] Mao A.H., Crick S.L., Vitalis A., Chicoine C.L., Pappu R.V. (2010). Net Charge per Residue Modulates Conformational Ensembles of Intrinsically Disordered Proteins. Proc. Natl. Acad. Sci. USA.

[35] Holehouse A.S., Garai K., Lyle N., Vitalis A., Pappu R.V. (2015). Quantitative Assessments of the Distinct Contributions of Polypeptide Backbone Amides versus Side Chain Groups to Chain Expansion via Chemical Denaturation. J. Am. Chem. Soc..

[36] Alvarez-Paggi D., Hannibal L., Castro M.A., Oviedo-Rouco S., Demicheli V., Tórtora V., Tomasina F., Radi R., Murgida D.H. (2017). Multifunctional Cytochrome c: Learning New Tricks from an Old Dog. Chem. Rev..

[37] Schweitzer-Stenner R., Hagarman A., Verbaro D., Soffer J.B. (2009). Conformational Stability of Cytochrome C Probed by Optical Spectroscopy. Methods Enzymol..

[38] Schwalbe H., Fiebig K.M., Buck M., Jones J.A., Grimshaw S.B., Spencer A., Glaser S.J., Smith L.J., Dobson C.M. (1997). Structural and Dynamical Properties of a Denatured Protein. Heteronuclear 3D NMR Experiments and Theoretical Simulations of Lysozyme in 8 M Urea. Biochemistry.

[39] Fitzkee N.C., Rose G.D. (2004). Reassessing Random-Coil Statistics in Unfolded Proteins. Proc. Natl. Acad. Sci. USA.

[40] Shi Z., Chen K., Liu Z., Ng A., Bracken W.C., Kallenbach N.R. (2005). Polyproline II Propensities from GGXGG Peptides Reveal an Anticorrelation with β-Sheet Scales. Proc. Natl. Acad. Sci. USA.

[41] Elam W.A., Schrank T.P., Hilser V.J., Schweitzer-Stenner R. (2012). Experimental and Computational Studies of Polyproline II Propensity. Protein and Peptide Folding, Misfolding, and Non-Folding.

[42] Hagarman A., Measey T.J., Mathieu D., Schwalbe H., Schweitzer-Stenner R. (2010). Intrinsic Propensities of Amino Acid Residues in GxG Peptides Inferred from Amide I’ Band Profiles and NMR Scalar Coupling Constants. J. Am. Chem. Soc..

[43] Hagarman A., Mathieu D., Toal S., Measey T.J., Schwalbe H., Schweitzer-Stenner R. (2011). Amino Acids with Hydrogen-Bonding Side Chains Have an Intrinsic Tendency to Sample Various Turn Conformations in Aqueous Solution. Chem.-A Eur. J..

[44] Schweitzer-Stenner R., Hagarman A., Toal S., Mathieu D., Schwalbe H. (2013). Disorder and Order in Unfolded and Disordered Peptides and Proteins: A View Derived from Tripeptide Conformational Analysis. I. Tripeptides with Long and Predominantly Hydrophobic Side Chains. Proteins Struct. Funct. Bioinform..

[45] Rybka K., Toal S.E., Verbaro D.J., Mathieu D., Schwalbe H., Schweitzer-Stenner R. (2013). Disorder and Order in Unfolded and Disordered Peptides and Proteins: A View Derived from Tripeptide Conformational Analysis. II. Tripeptides with Short Side Chains Populating Asx and β-Type like Turn Conformations. Proteins Struct. Funct. Bioinform..

[46] Meng W., Lyle N., Luan B., Raleigh D.P., Pappu R.V. (2013). Experiments and Simulations Show How Long-Range Contacts Can Form in Expanded Unfolded Proteins with Negligible Secondary Structure. Proc. Natl. Acad. Sci. USA.

[47] Lyle N., Das R.K., Pappu R.V. (2013). A Quantitative Measure for Protein Conformational Heterogneity. J. Chem. Phys..

[48] Das R.K., Pappu R.V. (2013). Conformations of Intrinsically Disordered Proteins Are Influenced by Linear Sequence Distributions of Appositely Charged Residues. Proc. Natl. Acad. Sci. USA.

[49] Salmon L., Nodet G., Ozenne V., Yin G., Jensen M.R., Zweckstetter M., Blackledge M. (2010). NMR Characterization of Long-Range Order in Intrinsically Disordered Proteins. J. Am. Chem. Soc..

[50] Harmon T.S., Holehouse A.S., Rosen M.K., Pappu R.V. (2017). Intrinsically Disordered Linkers Determine the Interplay between Phase Separation and Gelation in Multivalent Proteins. eLife.

[51] Ho B.K., Thomas A., Brasseur R. (2003). Revisiting the Ramachandran Plot: Hard-Sphere Repulsion, Electrostatics, and H-Bonding in the Alpha-Helix. Protein Sci..

[52] Ilawe N.V., Raeber A.E., Schweitzer-Stenner R., Toal S.E., Wong B.M. (2015). Assessing Backbone Solvation Effects in the Conformational Propensities of Amino Acid Residues in Unfolded Peptides. Phys. Chem. Chem. Phys..

[53] Fleming P.J., Fitzkee N.C., Mezei M., Srinivasan R., Rose G.D. (2009). A Novel Method Reveals That Solvent Water Favors Polyproline II over β-Strand Conformation in Peptides and Unfolded Proteins: Conditional Hydrophobic Accessible Surface Area (CHASA). Protein Sci..

[54] Lanza G., Chiacchio M.A. (2015). Interfacial Water at the Trialanine Hydrophilic Surface: A DFT Electronic Structure and Bottom-up Investigation. Phys. Chem. Chem. Phys..

[55] Diguiseppi D., Milorey B., Lewis G., Kubatova N., Farrell S., Schwalbe H., Schweitzer-Stenner R. (2017). Probing the Conformation-Dependent Preferential Binding of Ethanol to Cationic Glycylalanylglycine in Water/Ethanol by Vibrational and NMR Spectroscopy. J. Phys. Chem. B.

[56] Tanaka S., Scheraga H.A. (1976). Statistical Mechanical Treatment of Protein Conformation. 4. A Four-State Model for Specific-Sequence Copolymers of Amino Acids. Macromolecules.

[57] Tanaka S., Scheraga H.A. (1977). Statistical Mechanical Treatment of Protein Conformation. 5. A Multistate Model for Specific Sequence Copolymers of Amino Acids. Macromolecules.

[58] Karplus M. (1959). Theoretical Calculation Links NMR Coupling Constant to Molecular Geometry. J. Chem. Phys..

[59] Wang A.C., Bax A. (1996). Determination of the Backbone Dihedral Angles Φ in Human Ubiquitin from Reparametrized Empirical Karplus Equations. J. Am. Chem. Soc..

[60] Duddy W.J., Nissink J.W.M., Allen F.H., Milner-White E.J. (2008). Mimicry by Asx- and ST-Turns of the Four Main Types of β-Turn in Proteins. Protein Sci..

[61] Penkett C.J., Redfield C., Dodd I., Hubbard J., McBay D.L., Mossakowska D.E., Smith R.A.G., Dobson C.M., Smith L.J. (1997). NMR Analysis of Main-Chain Conformational Preferences in an Unfolded Fibronectin-Binding Protein. J. Mol. Biol..

[62] Hu J.S., Bax A. (1997). Determination of *φ* and χ_1_ Angles in Proteins from ^13^C-^13^C Three-Bond J Couplings Measured by Three-Dimensional Heteronuclear NMR. How Planar Is the Peptide Bond?. J. Am. Chem. Soc..

[63] Vuister G.W., Bax A. (1993). Quantitative J Correlation: A New Approach for Measuring Homonuclear Three-Bond J(HNHα) Coupling Constants in 15N-Enriched Proteins. J. Am. Chem. Soc..

[64] Wirmer J., Schwalbe H. (2002). Angular Dependence of 1J(Ni,Cαi) and 2J(Ni,Cα(i-1)} Couplings Constants Measured in J-Modulated HSQCs. J. Biomol. NMR.

[65] Hähnke M.J., Richter C., Heinicke F., Schwalbe H. (2010). The HN(COCA)HAHB NMR Experiment for the Stereospecific Assignment of H β-Protons in Non-Native States of Proteins. J. Am. Chem. Soc..

[66] Case D.A., Scheurer C., Bruschweiler R. (2000). Static and Dynamic Effects on Vicinal Scalar J Couplings in Proteins and Peptides: A MD/DFT Analysis. J. Am. Chem. Soc..

[67] Jha A.K., Colubri A., Zaman M.H., Koide S., Sosnick T.R., Freed K.F. (2005). Helix, Sheet, and Polyproline II Frequencies and Strong Nearest Neighbor Effects in a Restricted Coil Library. Biochemistry.

[68] Pappu R.V., Srinivasan R., Rose G.D. (2000). The Flory Isolated-Pair Hypothesis Is Not Valid for Polypeptide Chains: Implications for Protein Folding. Proc. Natl. Acad. Sci. USA.

[69] Ramachandran G.N., Ramakrishnan C., Sasisekharan V. (1963). Stereochemistry of Polypeptide Chain Configurations. J. Mol. Biol..

[70] Sosnick T.R. Sampling Library. http://godzilla.uchicago.edu/cgi-bin/rama.cgi.

[71] de Gennes P.-G. (1979). Scaling Concepts in Polymer Physics.

[72] Schweitzer-Stenner R. (2009). Distribution of Conformations Sampled by the Central Amino Acid Residue in Tripeptides Inferred from Amide i Band Profiles and NMR Scalar Coupling Constants. J. Phys. Chem. B.

[73] Schweitzer-Stenner R., Toal S.E. (2016). Construction and Comparison of the Statistical Coil States of Unfolded and Intrinsically Disordered Proteins from Nearest-Neighbor Corrected Conformational Propensities of Short Peptides. Mol. Biosyst..

[74] Tran H.T., Wang X., Pappu R.V. (2005). Reconciling Observations of Sequence-Specific Conformational Propensities with the Generic Polymeric Behavior of Denatured Proteins. Biochemistry.

[75] Gnanakaran S., Garcia A.E. (2003). Validation of an All-Atom Protein Force Field: From Dipeptides to Larger Peptides. J. Phys. Chem. B.

[76] Avbelj F., Baldwin R.L. (2004). Origin of the Neighboring Residue Effect on Peptide Backbone Conformation. Proc. Natl. Acad. Sci. USA.

[77] Baxa M.C., Haddadian E.J., Jha A.K., Freed K.F., Sosnick T.R. (2012). Context and Force Field Dependence of the Loss of Protein Backbone Entropy upon Folding Using Realistic Denatured and Native State Ensembles. J. Am. Chem. Soc..

[78] Zaman M.H., Shen M.Y., Berry R.S., Freed K.F., Sosnick T.R. (2003). Investigations into Sequence and Conformational Dependence of Backbone Entropy, Inter-Basin Dynamics and the Flory Isolated-Pair Hypothesis for Peptides. J. Mol. Biol..

[79] Toal S., Meral D., Verbaro D., Urbanc B., Schweitzer-Stenner R. (2013). PH-Independence of Trialanine and the Effects of Termini Blocking in Short Peptides: A Combined Vibrational, NMR, UVCD, and Molecular Dynamics Study. J. Phys. Chem. B.

[80] Peti W., Smith L.J., Redfield C., Schwalbe H. (2001). Chemical Shifts in Denatured Proteins: Resonance Assignments for Denatured Ubiquitin and Comparisons with Other Denatured Proteins. J. Biomol. NMR.

[81] Smith L.J., Bolin K.A., Schwalbe H., MacArthur M.W., Thornton J.M., Dobson C.M. (1996). Analysis of Main Chain Torsion Angles in Proteins: Prediction of NMR Coupling Constants for Native and Random Coil Conformations. J. Mol. Biol..

[82] Braun D., Wider G., Wüthrich K. (1994). Sequence-Corrected $^15$N “Random Coil” Chemical Shifts. J. Am. Chem. Soc..

[83] Peti W., Hennig M., Smith L.J., Schwalbe H. (2000). NMR Spectroscopic Investigation of ψ Torsion Angle Distribution in Unfolded Ubiquitin from Analysis of 3J(Cα,Cα) Coupling Constants and Cross-Correlated Γ(HNN1CαHα)/(C) Relaxation Rates. J. Am. Chem. Soc..

[84] MacKerell A.D., Bashford D., Bellott M., Dunbrack R.L., Evanseck J.D., Field M.J., Fischer S., Gao J., Guo H., Ha S. (1998). All-Atom Empirical Potential for Molecular Modeling and Dynamics Studies of Proteins. J. Phys. Chem. B.

[85] Ren P., Ponder J.W. (2002). Consistent Treatment of Inter- and Intramolecular Polarization in Molecular Mechanics Calculations. J. Comput. Chem..

[86] Mackerell A.D., Feig M., Brooks C.L. (2004). Extending the Treatment of Backbone Energetics in Protein Force Fields: Limitations of Gas-Phase Quantum Mechanics in Reproducing Protein Conformational Distributions in Molecular Dynamics Simulation. J. Comput. Chem..

[87] Kaminski G.A., Friesner R.A., Tirado-Rives J., Jorgensen W.L. (2001). Evaluation and Reparametrization of the OPLS-AA Force Field for Proteins via Comparison with Accurate Quantum Chemical Calculations on Peptides. J. Phys. Chem. B.

[88] Cruz V., Ramos J., Martínez-Salazar J. (2011). Water-Mediated Conformations of the Alanine Dipeptide as Revealed by Distributed Umbrella Sampling Simulations, Quantum Mechanics Based Calculations, and Experimental Data. J. Phys. Chem. B.

[89] Kwac K., Lee K.K., Han J.B., Oh K.I., Cho M. (2008). Classical and Quantum Mechanical/Molecular Mechanical Molecular Dynamics Simulations of Alanine Dipeptide in Water: Comparisons with IR and Vibrational Circular Dichroism Spectra. J. Chem. Phys..

[90] Kim Y.S., Wang J., Hochstrasser R.M. (2005). Two-Dimensional Infrared Spectroscopy of the Alanine Dipeptide in Aqueous Solution. J. Phys. Chem. B.

[91] Poon C.D., Samulski E.T., Weise C.F., Weisshaar J.C. (2000). Do Bridging Water Molecules Dictate the Structure of a Model Dipeptide in Aqueous Solution?. J. Am. Chem. Soc..

[92] Grdadolnik J., Grdadolnik S.G., Avbelj F. (2008). Determination of Conformational Preferences of Dipeptides Using Vibrational Spectroscopy. J. Phys. Chem. B.

[93] Oh K.-I., Jung Y.-S., Hwang G.-S., Cho M. (2012). Conformational Distributions of Denaturated and Unstructured Proteins Are Similar to Those of 20 × 20 Blocked Dipeptides. J. Biomol. NMR.

[94] Jung Y.S., Oh K.I., Hwang G.S., Cho M. (2014). Neighboring Residue Effects in Terminally Blocked Dipeptides: Implications for Residual Secondary Structures in Intrinsically Unfolded/Disordered Proteins. Chirality.

[95] He L., Navarro A.E., Shi Z., Kallenbach N.R. (2012). End Effects Influence Short Model Peptide Conformation. J. Am. Chem. Soc..

[96] Lee O., Roberts G.M., Diem M. (1989). IR Vibrational CD in Alanyl Tripeptide: Indication of a Stable Solution Conformer. Biopolymers.

[97] Ding L., Chen K., Santini P.A., Shi Z., Kallenbach N.R. (2003). The Pentapeptide GGAGG Has PII Conformation. J. Am. Chem. Soc..

[98] Chen K., Liu Z., Kallenbach N.R. (2004). The Polyproline II Conformation in Short Alanine Peptides Is Noncooperative. Proc. Natl. Acad. Sci. USA.

[99] Toal S.E., Kubatova N., Richter C., Linhard V., Schwalbe H., Schweitzer-Stenner R. (2015). Randomizing the Unfolded State of Peptides (and Proteins) by Nearest Neighbor Interactions between Unlike Residues. Chem. Eur. J..

[100] Milorey B., Schweitzer-Stenner R., Andrews B., Schwalbe H., Urbanc B. (2021). Short Peptides as Predictors for the Structure of Polyarginine Sequences in Disordered Proteins. Biophys. J..

[101] Milorey B., Schwalbe H., O’Neill N., Schweitzer-Stenner R. (2021). Repeating Aspartic Acid Residues Prefer Turn-like Conformations in the Unfolded State: Implications for Early Protein Folding. J. Phys. Chem. B.

[102] Makowska J., Rodziewicz-Motowidło S., Bagińska K., Vila J.A., Liwo A., Chmurzyński L., Scheraga H.A. (2006). Polyproline II Conformation Is One of Many Local Conformational States and Is Not an Overall Conformation of Unfolded Peptides and Proteins. Proc. Natl. Acad. Sci. USA.

[103] Zagrovic B., Lipfert J., Sorin E.J., Millett I.S., Van Gunsteren W.F., Doniach S., Pande V.S. (2005). Unusual Compactness of a Polyproline Type II Structure. Proc. Natl. Acad. Sci. USA.

[104] Shi Z., Anders Olson C., Rose G.D., Baldwin R.L., Kallenbach N.R. (2002). Polyproline II Structure in a Sequence of Seven Alanine Residues. Proc. Natl. Acad. Sci. USA.

[105] Tiffany M.L., Krimm S. (1968). New Chain Conformations of Poly(Glutamic Acid) and Polylysine. Biopolymers.

[106] Dukor R.K., Keiderling T.A. (1991). Reassessment of the Random Coil Conformation: Vibrational CD Study of Proline Oligopeptides and Related Polypeptides. Biopolymers.

[107] Makowska J., Rodziewicz-Motowidło S., Bagińska K., Makowski M., Vila J.A., Liwo A., Chmurzyński L., Scheraga H.A. (2007). Further Evidence for the Absence of Polyproline II Stretch in the XAO Peptide. Biophys. J..

[108] Sahoo H., Roccatano D., Hennig A., Nau W.M. (2007). A 10-Å Spectroscopic Ruler Applied to Short Polyprolines. J. Am. Chem. Soc..

[109] Rucker A.L., Pager C.T., Campbell M.N., Qualls J.E., Creamer T.P. (2003). Host-Guest Scale of Left-Handed Polyproline II Helix Formation. Proteins Struct. Funct. Genet..

[110] Mikhonin A.V., Myshakina N.S., Bykov S.V., Asher S.A. (2005). UV Resonance Raman Determination of Polyproline II, Extended 2.5 1-Helix, and β-Sheet ψ Angle Energy Landscape in Poly-L-Lysine and Poly-L-Glutamic Acid. J. Am. Chem. Soc..

[111] Schweitzer-Stenner R., Eker F., Griebenow K., Cao X., Nafie L.A. (2004). The Conformation of Tetraalanine in Water Determined by Polarized Raman, FT-IR, and VCD Spectroscopy. J. Am. Chem. Soc..

[112] McColl I.H., Blanch E.W., Hecht L., Kallenbach N.R., Barron L.D. (2004). Vibrational Raman Optical Activity Characterization of Poly(l-Proline) II Helix in Alanine Oligopeptides. J. Am. Chem. Soc..

[113] Verbaro D.J., Mathieu D., Toal S.E., Schwalbe H., Schweitzer-Stenner R. (2012). Ionized Trilysine: A Model System for Understanding the Nonrandom Structure of Poly- l -Lysine and Lysine-Containing Motifs in Proteins. J. Phys. Chem. B.

[114] Graf J., Nguyen P.H., Stock G., Schwalbe H. (2007). Structure and Dynamics of the Homologous Series of Alanine Peptides: A Joint Molecular Dynamics/NMR Study. J. Am. Chem. Soc..

[115] Verbaro D., Ghosh I., Nau W.M., Schweitzer-Stenner R. (2010). Discrepancies between Conformational Distributions of a Polyalanine Peptide in Solution Obtained from Molecular Dynamics Force Fields and Amide I′ Band Profiles. J. Phys. Chem. B.

[116] Schweitzer-Stenner R., Measey T.J. (2007). The Alanine-Rich XAO Peptide Adopts a Heterogeneous Population, Including Turn-like and Polyproline II Conformations. Proc. Natl. Acad. Sci. USA.

[117] Eker F., Cao X., Nafie L., Schweitzer-Stenner R. (2002). Tripeptides Adopt Stable Structures in Water. A Combined Polarized Visible Raman, FTIR, and VCD Spectroscopy Study. J. Am. Chem. Soc..

[118] Eker F., Griebenow K., Schweitzer-Stenner R. (2003). Stable Conformations of Tripeptides in Aqueous Solution Studied by UV Circular Dichroism Spectroscopy. J. Am. Chem. Soc..

[119] Rucker A.L., Creamer T.P. (2002). Polyproline II Helical Structure in Protein Unfolded States: Lysine Peptides Revisited. Protein Sci..

[120] Duitch L., Toal S., Measey T.J., Schweitzer-Stenner R. (2012). Triaspartate: A Model System for Conformationally Flexible DDD Motifs in Proteins. J. Phys. Chem. B.

[121] Schweitzer-Stenner R., Milorey B., Schwalbe H. (2022). Randomizing of Oligopeptide Conformations by Nearest Neighbor Interactions between Amino Acid Residues. Biomolecules.

[122] Oh K.-I., Lee K.-K., Park E.-K., Jung Y.-S., Hwang G.-S., Cho M. (2012). A Comprehensive Library of Blocked Dipeptides Reveals Intrinsic Backbone Conformational Propensities of Unfolded Proteins. Proteins Struct. Funct. Genet..

[123] Lumry R., Shyamala R. (1970). Enthalpy-Entropy Compensation Phenomena in Water Solutions of Proteins and Small M Olecules: A Ubiquitous Property of Water. Biopolymers.

[124] Dunitz J.D. (1995). Win Some, Lose Some: Enthalpy-Entropy Compensation in Weak Intermolecular Interactions. Chem. Biol..

[125] Liu L., Guo Q.X. (2001). Isokinetic Relationship, Isoequilibrium Relationship, and Enthalpy-Entropy Compensation. Chem. Rev..

[126] Toal S.E. (2014). Defining Local Order in the Unfolded State Using Short Peptide Model Systems and Spectroscopic Methods: Conformational Biases, Meditation by Solvation and Nearest Neighbor Effects. Ph.D. Thesis.

[127] Schweitzer-Stenner R., Toal S.E. (2018). Anticooperative Nearest-Neighbor Interactions between Residues in Unfolded Peptides and Proteins. Biophys. J..

[128] Vitalis A., Pappu R.V. (2009). ABSINTH: A New Continuum Solvation Model for Simulations of Polypeptides in Aqueous Solutions. J. Comput. Chem..

[129] Dill K.A. (1997). Additivity Principles in Biochemistry. J. Biol. Chem..

[130] Andrews B., Guerra J., Schweitzer-Stenner R., Urbanc B. (2022). Do Molecular Dynamics Force Fields Accurately Model Ramachandran Distributions of Amino Acid Residues in Water?. Phys. Chem. Chem. Phys..

[131] Wright P.E., Dyson H.J. (1999). Intrinsically Unstructured Proteins: Re-Assessing the Protein Structure-Function Paradigm. J. Mol. Biol..

[132] Dyson H.J., Wright P.E. (1993). Peptide Conformation and Protein Folding. Curr. Opin. Struct. Biol..

[133] Dyson H.J., Wright P.E. (1991). Defining Solution Conformations of Small Linear Peptides. Annu. Rev. Biophys. Biophys. Chem..

[134] Zimm B.H., Bragg J.K. (1959). Theory of the Phase Transition between Helix and Random Coil in Polypeptide Chains. J. Chem. Phys..

[135] Lifson S., Roig A. (1961). On the Theory of Helix-Coil Transitions in Polypeptides. J. Chem. Phys..

